# Impact of content features of digital micro-dramas on active aging: the serial mediation effect of emotional engagement and social connectedness

**DOI:** 10.3389/fpsyg.2025.1711672

**Published:** 2025-11-07

**Authors:** Wei Li, Shijing Cheng, Shuai Ling, Can Zheng

**Affiliations:** 1 Zhang Daqian School of Fine Arts, Neijiang Normal University, Neijiang, China; 2 School of Ceramic Art and Design Art, Jingdezhen University, Jingdezhen, China; 3 College of Art and Design, Nanjing Forestry University, Nanjing, China; 4 School of Media, Jiangsu Second Normal University, Nanjing, China

**Keywords:** digital micro-dramas, content characteristics, active aging, emotional engagement, social connectedness

## Abstract

Due to the rapid development of digital media, micro-dramas have emerged as an important channel for cultural consumption and social participation among the elderly due to their concise narrative, emotional intensity, and convenient interactivity. This study leverages the theory of active aging to explore the impact mechanisms of the content characteristics of digital micro-drama (narrativity, emotional resonance, and interactivity) on active aging among the elderly, introducing emotional engagement and social connectedness as mediating variables. The study employs a questionnaire survey and structural equation modeling method to conduct an empirical analysis on a sample of 705 elderly individuals from urban China. The results indicate that the narrativity, emotional resonance, and interactivity of digital micro-dramas positively influence the level of active aging; emotional engagement demonstrates partial mediation in this process; and social connectedness plays a mediating role in several pathways. Furthermore, emotional engagement and social connectedness may exert a chain mediation effect, further strengthening the potential impact of micro-dramas on active aging. This study reveals the role of digital micro-dramas, as an emerging cultural form, in promoting psychological health and social participation among the elderly, providing theoretical support and practical insights for advancing the active aging strategy and digital cultural services for the elderly.

## 1 Introduction

Population aging has emerged as one of the most significant demographic changes of the 21st century, with the proportion of elderly populations rapidly increasing in both developed and developing countries (Nations, U, 2013; China: National Bureau of Statistics of China, 2021). According to the World Health Organization (WHO), the proportion of the global population aged 60 and above will nearly double between 2015 and 2050, growing from 12 to 22% (World Health Organization, 2023). Therefore, addressing the aging problem has become a global priority. Against this backdrop, active aging has been proposed as a holistic perspective, emphasizing the importance of maintaining physical health, psychological well-being, and active social participation during later years (Havighurst, 1961; Rowe and Kahn, 1997). According to the theory of aging activities, older adults can improve life satisfaction and slow functional decline by continuously participating in social, cognitive, and physical activities (Bowling, 2008).

In the internet era, the widespread adoption of digital media has provided unprecedented opportunities for older adults to maintain social connectedness and emotional vitality (Chen and Schulz, 2016; Cotten et al., 2013). According to a survey by the Pew Research Center, as of 2024, approximately 90% of adults aged 65 and above use the internet (Results, 2025). In China, by 2022, over 152 million people aged 60 and above were using the internet, accounting for 14.3% of all internet users (Zhou et al., 2024). Among them, 70.7% use mobile payment (Wu, 2024), 46.5% watch short videos daily, and 63.4% engage in digital social interactions (Cui et al., 2024). A large body of empirical research has shown that digital engagement can markedly alleviate symptoms of depression, enhance cognitive function, and improve the quality of life in later years (Choi et al., 2012; Chopik, 2016; Liu and Li, 2024). For example, data from the Health and Retirement Study in the United States indicated that digital social media communication can enhance perceived social support, thereby reducing feelings of loneliness and promoting subjective well-being (Zhang et al., 2021).

Digital micro-dramas (i.e., narrative short videos typically lasting no longer than 5 min) have been widely adopted by the elderly to promote social connectedness, enhance memory, and foster a sense of self-identity, highlighting the potential appeal of this medium for aging populations (Rios Rincon et al., 2022). Micro-dramas have gradually become part of the daily lives of older adults. According to WIRED, over 267 million elderly individuals in China use Douyin (the Chinese version of TikTok)(Higgins, 2023). In recent years, elderly content creators have also begun to emerge on short video platforms, reflecting a significant increase in the participation and visibility of older users within the digital media ecosystem (Tang et al., 2023). Micro-dramas are characterized by concise narratives, strong emotional resonance, and rich interactive features, which align with the viewing habits and cognitive preferences of elderly users (Gao et al., 2024; Qin, 2024). According to narrative gerontology, telling life stories can enhance self-identity, psychological resilience, and a sense of meaning in later life (Bohlmeijer et al., 2007; Stargatt et al., 2022). Related studies have also indicated that digital storytelling can improve emotional health among the elderly, enhance positive emotions, promote social participation, and contribute to active aging (Rios Rincon et al., 2022; Xu et al., 2023). The frequency of social media use in elderly populations with smaller social network scales has been found to have a significant negative correlation with negative emotions, suggesting that social media, to some extent, serves as a “compensatory social resource”(Kim and Fingerman, 2022). Additionally, media interactivity is considered a key feature behind user communication and engagement (Liu et al., 2022). For example, the interactive features in micro-dramas (such as comment sections, audience voting, and user-created derivative content) provide an important channel for fostering a sense of community and mutual recognition among viewers (Chen, 2024). Therefore, the rising popularity of digital micro-dramas among the elderly not only reflects their entertainment function but also highlights their unique value in promoting emotional health, social connectedness, and self-identity.

When exploring the relationship between digital media and elderly populations, it is important not only to focus on the instrumental functions and social participation aspects but also to recognize the core role of emotional engagement in social connectedness. According to the socioemotional selectivity theory (SST), as individuals age, their perception of the future becomes more constricted, leading them to prioritize emotional fulfillment and positive social interactions over mere information acquisition or social expansion (Carstensen, 1992). Digital media align with the motivational shift mechanism revealed by SST, offering immediate emotional satisfaction, low-barrier social interaction, and enhanced belonging, which meet the elderly’s core needs for positive emotions and social connectedness in later life (Nimrod, 2020b; Quan-Haase et al., 2017). Meanwhile, emotional engagement plays a significant role in fostering social connectedness and psychological health. Research has shown that emotional media experiences can stimulate more social interactions, thereby strengthening social support and interpersonal ties (Nabi and Myrick, 2019; Vorderer et al., 2004). Therefore, from the perspective of both the theories of active aging and socioemotional selectivity, emotional engagement is arguably not only an ancillary effect of digital media use but also a core mechanism for elderly individuals to maintain social connectedness, psychological health, and overall well-being.

Although scholarly attention has increasingly been focusing on digital media, existing research has focused on its impact on information acquisition, health management, and cognitive stimulation among elderly populations (Chai et al., 2024; Cotten et al., 2022). For example, some studies have found that internet use helps older adults access health and cultural information, improve attention and motor skills, and enhance cognitive reserves by alleviating depressive symptoms (Chen et al., 2024). Moreover, longitudinal studies have shown that elderly individuals who use the internet frequently are at lower risk of cognitive decline and have higher health literacy (Yu et al., 2022). Digital technology interventions have also demonstrated substantial improvements to attention, memory, and executive function in older adults (Chen et al., 2025). However, systematic research on how digital micro-dramas promote social connectedness and psychological health in the elderly through emotional engagement remains scarce. Existing studies have indicated that the use of digital social media can reduce feelings of loneliness and social isolation among older adults.(Liu and Li, 2024) Notwithstanding, its emotional engagement mechanisms remain underexplored. Moreover, in-depth empirical research on how the content characteristics of digital micro-dramas, such as interactivity, emotional resonance, and narrativity, influence elderly social connectedness and psychological health through emotional engagement is lacking. Therefore, investigating the mechanism by which emotional engagement in digital micro-drama content characteristics affects social connectedness and psychological health in the elderly has important theoretical and practical significance.

To further strengthen the theoretical foundation of this study, three content characteristics, namely narrativity, emotional resonance, and interactivity, were selected as key independent variables. These three elements constitute the core mechanisms through which digital narratives influence audience cognition and emotion. According to media psychology theory, narrative promotes cognitive immersion and meaning construction (Green and Brock, 2000), emotional resonance stimulates affective empathy and reflective thinking (Nabi and Green, 2014), and interactivity enhances participatory communication through real-time feedback and social presence (Sundar and Limperos, 2013). These three characteristics collectively reveal the integrated effects of digital micro-dramas across cognitive, emotional, and behavioral dimensions. Furthermore, this study employs emotional engagement and social connectedness as chained mediating variables, grounded in positive aging theory (Rowe and Kahn, 1997) and the social–emotional selection theory (Carstensen, 1992). Emotional engagement serves as a proximal psychological mechanism, reflecting the transformation of media experiences into emotional responses. In contrast, social connectedness serves as the distal social outcome, further transforming emotional engagement into social participation and well-being. The relationship between these two is supported by the theory of emotional contagion (Barsade, 2002) and the model of emotional social sharing (Rimé, 2009), which indicate that the social sharing of emotional experiences can enhance interpersonal bonds and social support. Therefore, the chained mediation model proposed in this study has a solid theoretical foundation. (Reviewer2-1).

Based on the above theories, this study aims to answer the following research questions:

1. How do the content characteristics of digital micro-dramas (narrativity, emotional resonance, and interactivity) influence the level of active aging in older adults?
2. What is the mediating role of emotional engagement between the content characteristics of digital micro-dramas and active aging in older adults?
3. What is the mediating role of social connectedness in the impact of digital micro-drama content characteristics on active aging in older adults?

To address the above questions, this study constructs a research model of digital micro-drama content characteristics: emotional engagement–social connectedness–active aging. SPSS 26 was used to perform descriptive statistical analysis of the questionnaire data, and SmartPLS 4 was employed to test the model and reveal the relationships between the variables. By integrating perspectives from media psychology, gerontology, and communication studies, this research not only fills gaps in the existing literature but also provides empirical references and practical recommendations for designing culturally adaptive and emotionally resonant digital media content for older adults.

## 2 Research hypotheses and theoretical framework

### 2.1 Theoretical framework

#### 2.1.1 Digital micro-drama content characteristics and active aging

Digital micro-dramas have rapidly emerged in recent years as a short, fast-paced, and tightly structured form of visual content on social media and video platforms, typically with an episode length of 1–5 min. They maintain audience engagement through continuous updates and progressive plot development (Reuters, 2024). These dramas often focus on everyday-life scenarios, such as family, neighborhood, health, and re-employment, combining emotional climaxes and resonance points to satisfy viewers’ emotional needs and enhance interactivity. This gives them wide appeal, especially among middle-aged and elderly audiences (Huang and Sun, 2011; Junxun, 2024; Yuan, 2024). By depicting relatable life situations and emotional tension, digital micro-dramas evoke empathy and emotional engagement from the viewers. Audiences not only resonate with the content but also tend to engage in social interactions through comments and shares, expanding their social connectedness and sense of belonging (Cohen, 2001; Green and Brock, 2000). Studies have shown that, with the proliferation of the internet and mobile media among the elderly, digital media can offer them “emotional compensation” and a “sense of social connectedness”(Sen et al., 2021). In East Asian contexts, including on Chinese video platforms, real-time synchronized comments (bullet comments) and timeline annotations transform “solo viewing” into a “shared experience,” effectively enhancing emotional resonance and group identity. This “micro-interaction” based on comments, likes, and bullet comments further boosts the sense of social presence and immersion, thus strengthening community belonging (Garcia et al., 2016; Zhou and Zhou, 2022). It can directly expand the weak-tie networks of the elderly, which aligns with the participation dimension of active aging (Chopik, 2016).

The WHO defines active aging as “the process of optimizing opportunities for health, participation, and security to enhance the quality of life in older age.” This multidimensional framework emphasizes not only physical health but also the ongoing participation of older adults in social, cultural, and public life (Paúl et al., 2012). Under the active aging framework, digital micro-dramas may serve as an important mediating variable in promoting the psychological health, social participation, and life satisfaction of the elderly population (Zhang et al., 2024). The densely packed plots and intense emotional design of micro-dramas can rapidly induce immersion and emotional peaks. Empirical research has shown that narrative content significantly increases audience engagement and willingness to share (Green et al., 2004; Kang et al., 2020). Accordingly, micro-dramas with positive themes or emotional resonance are more likely to evoke positive emotions in elderly viewers, encouraging them to share and discuss the storyline with others, thereby strengthening social connections (Zhang et al., 2024). According to social science research, strong social relationships significantly reduce the risk of mortality and improve physical and mental health. In contrast, feelings of loneliness increase the risks of heart disease, cognitive decline, and early death (House et al., 1988; Rook, 2015). Therefore, enhancing social connectedness and emotional engagement in the elderly through digital micro-dramas not only aligns with the “participation” and “security” dimensions of active aging but also potentially holds public health significance.

Building on existing research, the core content characteristics of micro-dramas can be further understood through three dimensions: narrativity, emotional resonance, and interactivity. First, narrativity is a fundamental attribute of audiovisual works. Narrative studies have indicated that stories with a complete structure and clear causality can enhance the viewer’s immersive experience and psychological involvement. This allows older adults to engage in “meaning-making,” namely, reflecting on their personal lives and achieving a sense of satisfaction (Green and Brock, 2000; Polkinghorne, 1991). Empirical research has also demonstrated that content with strong narrativity significantly boosts the audience’s tendency to think about and share the content (Igartua and Vega Casanova, 2016). Second, emotional resonance is a key driver of continuous viewer engagement. According to theories on emotion, emotional responses can directly influence individual attitudes and social interactions (Lazarus, 1991). For elderly groups, emotional expressions related to family, intergenerational communication, and similar themes can evoke strong emotional reactions, alleviating feelings of loneliness and increasing social support (Nabi and Green, 2014). Positive emotions also expand an individual’s social network, increasing opportunities for social participation (Fredrickson, 2001). Finally, digital media, in contrast to traditional audiovisual works, are characterized by interactivity. According to the uses and gratifications theory (Trowbridge, 1976), interactive features satisfy individuals’ need for self-expression and provide immediate social feedback. Studies have shown that “micro-interactions” based on comments, likes, and bullet comments can substantially enhance the viewer’s sense of social presence and group belonging (Wohn et al., 2011). For the elderly, such interactions not only break the limitations of offline socializing but also expand weak-tie networks and social capital (Zuo and Wang, 2023), further promoting active aging. In summary, this paper argues that the content characteristics of digital micro-dramas should be studied from the perspectives of narrativity, emotional resonance, and interactivity.

This study predicts that when micro-dramas feature stronger narrativity, higher emotional resonance, and richer interactive functions, older adults will exhibit improved psychological health, social participation, and life satisfaction—the core goals of active aging. Therefore, this study proposes the following hypotheses:

Hypothesis 1a (H1a): The narrativity of digital micro-dramas positively influences the level of active aging in elderly individuals.

Hypothesis 1b (H1b): The emotional resonance of digital micro-dramas positively influences the level of active aging in elderly individuals.

Hypothesis 1c (H1c): The interactivity of digital micro-dramas positively influences the level of active aging in elderly individuals.

#### 2.1.2 Digital micro-drama content characteristics and emotional engagement

Emotional engagement is the emotional investment, emotional arousal, and inner resonance experienced by individuals during media consumption, with its core being the emotional connectedness established between the user and the media content (Hollebeek et al., 2014; Nabi and Green, 2014). In the digital environment, short-duration, high-density narrative forms, such as digital micro-dramas, have become an important medium for promoting emotional engagement due to their narrativity, emotional resonance, and interactivity.

Narrativity enhances emotional engagement through “narrative transportation” and “narrative involvement.” The theory of narrative transportation suggests that when individuals become immersed in a well-structured and engaging narrative, their attention, emotions, and cognition are “transported” into the story context, thereby enhancing emotional experiences and altering attitudes and behaviors (Green and Brock, 2000; Gerrig, 1993). Further, the narrative involvement model emphasizes that narrative coherence, character understanding, and mental imagery collectively shape the audience’s emotional participation (Busselle and Bilandzic, 2009; Slater and Rouner, 2002). Digital micro-dramas, 1–5 min long, rapidly achieve immersion through “hooked” openings, conflict tension, and emotional twists, which aligns with existing research that has indicated that even short narratives can trigger marked transportation and emotional responses (Igartua and Vega Casanova, 2016; Laer et al., 2014). Emotional resonance is a key driver of sustained participation. According to appraisal–arousal-oriented emotion theory, content that triggers high-arousal positive emotions, such as warmth, joy, and admiration, is more likely to shorten psychological distance and amplify the intensity of participation (Nabi and Green, 2014; Scherer, 2001). Discrete emotion research and the emotional persuasion model have suggested that empathy and character identification induced by narratives increase attention, memory, and willingness to share (Cohen, 2001; Nabi and Green, 2014). Platform data and empirical studies have also found that content that triggers high-arousal positive emotions is more likely to be shared and discussed (Berger and Milkman, 2012). For elderly populations, this mechanism aligns closely with SST: as their perception of future time narrows, older adults tend to prefer emotionally significant and intimate experiences (Carstensen et al., 1999; Carstensen et al., 2006). Therefore, micro-dramas focusing on family, intergenerational interaction, and elderly life are more likely to evoke emotional engagement and sharing. Interactivity enhances and amplifies emotional engagement through social presence, agency, and social sharing. The MAIN (Modality, Agency, Interactivity, Networkedness) model posits that the modalities (Modality), agency (Agency), interactivity as a source of agency (Interactivity), and networkedness (Networkedness) of digital media collectively shape the psychological heuristics for participation (Sundar, 2008a; Sundar and Limperos, 2013). “Micro-interactions,” such as bullet comments, reviews, and likes, create immediate emotional co-presence and feedback during viewing, enhancing social presence and belonging (Lombard and Ditton, 1997; Short et al., 1976). Additionally, social sharing effects extend emotions (Rimé, 2009). Relevant studies have also shown that interactive features on OTT platforms enhance emotional engagement and platform stickiness (Ajith et al., 2025), whereas character identification and para-social relationships further promote emotional participation (Eyal and Cohen, 2006; Tukachinsky and Stever, 2019).

In summary, narrativity enhances emotional arousal through narrative transportation and involvement, emotional resonance strengthens emotional engagement through discrete positive emotions and empathy, and interactivity maintains and amplifies emotional involvement through social presence and social sharing. Therefore, this study proposes the following hypotheses:

Hypothesis 2a (H2a): The narrativity of digital micro-dramas has a significant positive impact on emotional engagement.

Hypothesis 2b (H2b): The emotional resonance of digital micro-dramas has a significant positive impact on emotional engagement.

Hypothesis 2c (H2c): The interactivity of digital micro-dramas has a significant positive impact on emotional engagement.

#### 2.1.3 Digital micro-drama content characteristics and social connectedness

Social connectedness is typically defined as an individual’s subjective experience of connection and belonging with others, groups, and society and serves as an important psychosocial resource for health and well-being in later life. This concept is often operationalized in psychometrics using the Social Connectedness Scale, emphasizing the sustained experience of “being connected/accepted”(Lee and Robbins, 1995). Existing research has shown that both the quantity and quality of social relationships are closely associated with mortality risk, mental health, and life quality (Holt-Lunstad et al., 2010; National Academies of Sciences and Medicine, 2020). The social integration–health model further suggests that social networks, through psychological mechanisms (such as belonging, meaning, and perceived support), and physiological pathways (such as stress responses and inflammation) collectively influence health outcomes (Berkman et al., 2000; Cohen and Wills, 1985; Uchino, 2006). Therefore, any media content and mechanisms that promote social connectedness are particularly meaningful for elderly individuals.

Mechanistically, narrative transportation and immersion allow viewers to quickly “enter the story world,” fostering shared situational experiences and character identification. This process enhances empathy and social processing, which are associated with stronger social connectedness and a better understanding of others (Busselle and Bilandzic, 2009; Green and Brock, 2000; Laer et al., 2014). Research has indicated that character identification and empathy, triggered by narratives, help improve prosocial attitudes and interpersonal understanding (Johnson, 2012; Mar and Oatley, 2008). The rapid immersion and densely packed plots of digital micro-dramas stimulate user interaction within social networks, thus enhancing social capital and promoting the formation of shared conversation topics and narrative cues in family chats and social circles. This contributes to the generation and consolidation of bridging and bonding social capital (Ellison et al., 2007; Putnam, 2000). Regarding the mechanism underlying social connectedness, emotional resonance is not only an important precondition for emotional engagement but also key to maintaining social relationships. Emotional resonance can promote group cohesion and social belonging through emotional synchrony and shared affective experiences (Barsade, 2002; Páez et al., 2015). Emotional contagion theory posits that positive emotions triggered by media content spread through social interactions, fostering prosocial interactions and social connectedness (Hatfield et al., 1993). In the context of micro-dramas, this resonance is often triggered by narratives centered around family, affection, and intergenerational interaction. As a result, the psychological distance between the audience and the characters is shortened, which provides an emotional “common language” for subsequent social exchanges. Interactivity is the key content feature that connects these psychological processes with social outcomes. Media interactivity activates social motivations through perceived responsiveness and bidirectionality (Liu and Shrum, 2002). The MAIN model and social presence theory suggest that when users experience a sense of “others being present,” relational participation and social exchange are enhanced (Lombard and Ditton, 1997; Sundar, 2008b; Sundar and Limperos, 2013). In Eastern platforms, bullet comments and on-screen comments transform solitary viewing into a shared viewing experience, substantially improving social presence, belonging, and sustained engagement. These micro-interactions can rapidly create small connections during short viewing sessions (Hamilton et al., 2014; Hilvert-Bruce et al., 2018). Such interactive mechanisms align with the evidence from information and communication technology (ICT) interventions for the elderly. ICT interventions targeting older adults can alleviate social isolation, enhance social support, and stimulate interest in participation in the short term (Chen and Schulz, 2016; Leist, 2013). The use of digital social technologies by elderly individuals is associated with better subjective health, less depression, and higher life satisfaction (Chopik, 2016; Heo et al., 2015; Nowland et al., 2018).

In summary, the narrativity of digital micro-dramas promotes empathy and social understanding through narrative transportation and character identification (Busselle and Bilandzic, 2009; Green and Brock, 2000; Johnson, 2012; Laer et al., 2014; Mar and Oatley, 2008). Likewise, emotional resonance strengthens social sharing and relationship maintenance through emotional synchrony and emotional contagion (Barsade, 2002; Hatfield et al., 1993; Páez et al., 2015). Finally, interactivity, by relying on social presence and real-time feedback, transforms into group-based interaction, thereby enhancing social connectedness and a sense of belonging (Chen and Schulz, 2016; Hilvert-Bruce et al., 2018; Liu and Shrum, 2002; Lombard and Ditton, 1997; Nowland et al., 2018; Sundar, 2008a; Sundar and Limperos, 2013). Accordingly, this study proposes the following hypotheses:

Hypothesis 3a (H3a): The narrativity of digital micro-dramas has a significant positive impact on social connectedness in elderly individuals.

Hypothesis 3b (H3b): The emotional resonance of digital micro-dramas has a significant positive impact on social connectedness in elderly individuals.

Hypothesis 3c (H3c): The interactivity of digital micro-dramas has a significant positive impact on social connectedness in elderly individuals.

#### 2.1.4 Emotional engagement and active aging

Emotional engagement is widely recognized as an important psychological mechanism for active aging, promoting elderly individuals’ mental health, social participation, and overall life satisfaction (Ryff and Singer, 2008; Yen et al., 2022). SST suggests that, as the perception of future time shortens, older adults tend to prioritize activities and relationships with emotional significance to optimize positive emotional experiences and enhance well-being (Carstensen, 1992; Carstensen et al., 1999). This selective emotional engagement not only reduces negative emotional experiences but also strengthens psychological resilience, playing an adaptive role in the process of active aging (Carstensen, 2006).

In the context of digital media, the forms of emotional engagement are becoming increasingly diversified. Research has indicated that digital content that evokes strong emotional reactions in older adults not only helps enhance their sense of meaning in life but also increases their willingness to engage in social activities (Fredrickson, 2004; M and Blanchard-Fields, 2012). This process aligns with Fredrickson’s broaden-and-build theory, which suggests that positive emotions can expand an individual’s thinking and action reserves and, gradually, build lasting psychological and social resources. In other words, emotional engagement can transform brief emotional experiences into long-term social integration and personal growth resources (Hughes et al., 2008). Moreover, a large body of empirical research has demonstrated that emotional engagement has a positive impact on elderly individuals’ health behaviors. Positive emotional states not only increase compliance with exercise, dietary management, and chronic disease self-regulation but also reduce feelings of loneliness and depression (Chopik, 2016; Pressman and Cohen, 2005). Additionally, emotional engagement obtained through digital leisure and interaction can enhance elderly individuals’ social identity, sense of belonging, and collective efficacy, all of which are considered important psychosocial drivers of active aging (Barbosa Neves et al., 2017; Liu and Li, 2024).

In summary, emotional engagement, as a core psychological driver of active aging, operates multi-dimensionally, spanning emotional regulation, cognitive maintenance, social interaction, and health behaviors. In the context of emerging media, such as digital micro-dramas, emotional engagement not only enhances elderly individuals’ immediate emotional experiences but also promotes social connectedness and strengthens psychological capital. As such, the following hypotheses are proposed:

Hypothesis 4 (H4): Emotional engagement has a significant positive impact on active aging in elderly individuals.

#### 2.1.5 Social connectedness and active aging

Social connectedness is a critical pillar of active aging. It provides emotional support and enhances psychological health and life satisfaction through interaction (Haslam et al., 2018). In its framework for active aging, the WHO explicitly states that health, participation, and security are the three pillars for promoting successful aging, with social participation and social support being key pathways to achieving these goals (World Health Organization, 2002). Research has shown that social connectedness not only improves the psychological health, life satisfaction, and overall well-being of older adults but also significantly reduces disease risk and delays functional decline through behavioral, psychological, and physiological mechanisms (Holt-Lunstad, 2022). Stable and rich social ties can improve the health levels and quality of life of elderly individuals by buffering stress, providing emotional and instrumental support, promoting healthy behaviors, and enhancing cognitive stimulation (Cohen and Wills, 1985).

Social identity and the perspective of “social healing” highlight the importance of group belonging and shared identity. By enhancing individuals’ sense of belonging, self-worth, and collective support, this group embedding not only alleviates feelings of loneliness but also significantly improves psychological health and social participation (Haslam et al., 2018). Strengthening social identity through collective embedding enhances an individual’s social capital, giving elderly individuals a more solid sense of security and belonging and allowing them to adapt better to age-related challenges (Cruwys et al., 2013). Meanwhile, the expansion of social networks can also enhance information exchange, the ability to access social resources, cultural participation, and intergenerational interaction, all of which influence active aging (Panzarasa et al., 2020). In the digital context, online social tools provide new dimensions and opportunities for social connectedness among older adults. Empirical studies have shown that the active use of social media, video calls, and other online communication tools by elderly individuals is significantly correlated with reduced feelings of loneliness, improved subjective well-being, and better self-rated health. This effect is mainly achieved through the reduction of loneliness and the enhancement of social support (Chopik, 2016). Digital platforms not only offer cross-space social interactions but also strengthen social belonging through interaction, sharing, and emotional resonance, enabling elderly individuals to receive psychological and social resource support even in virtual environments (Gao et al., 2024). Further research has indicated that the combination of structural support (such as community participation opportunities) and functional support (such as perceived support and belonging) can maximize the protective effect of social connectedness on elderly health and well-being (Chen and Zhang, 2022; Iqbal et al., 2025).

In summary, social connectedness exerts a significant influence on active aging through multiple pathways. On one hand, it alleviates psychological stress and reduces loneliness by providing emotional and instrumental support. On the other hand, it enhances well-being and life satisfaction by strengthening group belonging and social identity. Moreover, digital social tools further expand the space and opportunities for social participation, providing an important avenue for elderly individuals to gain social capital and psychological resources. Based on this, the following research hypotheses are proposed:

Hypothesis 5 (H5): Social connectedness has a significant positive impact on active aging in elderly individuals.

#### 2.1.6 Emotional engagement and social connectedness

Emotional engagement refers to the emotional investment and resonance that individuals experience during media consumption. It is a crucial psychological mechanism for facilitating social connectedness (Vorderer et al., 2004). Emotional engagement is not only an internal psychological experience but also promotes interpersonal social connectedness by stimulating empathy, emotional resonance, and shared memories (Nabi and Green, 2014). In digital narrative environments, such as short videos and micro-dramas, viewers’ emotional responses are often characterized by strong emotional reactions to the plot and characters. This emotional investment can transcend the boundaries between reality and the virtual world, enhancing viewers’ willingness to interact and their sense of social connectedness (Slater et al., 2014). This phenomenon aligns with the theory of narrative immersion, which emphasizes that emotional media experiences encourage viewers to deeply immerse themselves in characters and social contexts, thereby improving interpersonal understanding and relationship building (Green and Brock, 2000).

Psychological research has revealed two primary pathways through which emotional engagement facilitates social connectedness. The first is the emotional resonance pathway, where viewers experience emotional synchronization during media consumption, thereby strengthening group belonging and social identity (Cheong et al., 2023; Decety and Jackson, 2004). Studies have shown that emotional synchronization and shared emotional experiences significantly enhance group cohesion and social trust (Nummenmaa et al., 2012). The second pathway is social sharing, where Rimé’s theory of emotional social sharing suggests that individuals tend to share their emotional experiences with others, either face-to-face or on online platforms. This sharing not only spreads emotions but also facilitates the formation of social support networks (Rimé, 2009). Existing research has indicated that when media content evokes emotional resonance, it often triggers more social interactions, thereby increasing social capital and interpersonal connections (Oh et al., 2014). In elderly populations, the relationship between emotional engagement and social connectedness is particularly important. As individuals age, their social networks often shrink due to retirement, physical decline, or the loss of a spouse, making elderly people more reliant on emotional interactions to maintain social connectedness (Carstensen et al., 1999). Studies have shown that positive emotional experiences can alleviate feelings of loneliness and isolation, thereby enhancing psychological health and life satisfaction in older adults (Pinquart and Sörensen, 2000). Positive emotional experiences gained through digital media not only help maintain existing social relationships but also facilitate the establishment of new social bonds (Nimrod, 2020a). Furthermore, emotional media content often revolves around themes of family, friendship, and intergenerational relationships, providing common ground for older adults and younger generations. This becomes an important bridge for cross-generational communication and social cohesion (Williams and Nussbaum, 2013). Empirical studies have also revealed that, after engaging in emotionally interactive digital activities, elderly individuals report a significant increase in subjective well-being and sense of social belonging (Chopik, 2016).

In summary, in the context of digital micro-dramas, emotional engagement is not only an aesthetic experience but also a crucial psychological driver of social connectedness and social capital. It fosters social integration within the elderly population through emotional resonance and social sharing mechanisms and plays a bridging role in intergenerational communication (Putnam, 2000). Therefore, this study proposes the following hypotheses:

Hypothesis 6 (H6): Emotional engagement has a significant positive impact on social connectedness in elderly individuals.

#### 2.1.7 Mediating effect of emotional engagement on social connectedness

Based on the previous hypotheses, it is suggested that the narrativity of digital micro-dramas enhances emotional arousal, emotional resonance strengthens emotional engagement by triggering empathy, and interactivity maintains emotional involvement through social feedback and presence, thereby increasing the level of active aging (Hypotheses H2a, H2b, H2c). The sequential relationship between emotional involvement and social connectedness can be explained by the theory of emotional contagion and the model of emotional social sharing (Rimé, 2009). Emotional involvement during media exposure triggers affective resonance and emotional synchrony, thereby promoting interpersonal communication and social understanding (Nummenmaa et al., 2012). When individuals develop strong emotional engagement with narrative content, they are more inclined to share this experience, which enhances relational intimacy and group identification (Barsade, 2002; Nabi and Myrick, 2019). Among older adults, this “emotion–society” pathway aligns with the social emotion selection theory (Carstensen et al., 1999), which posits that positive emotional experiences are crucial in maintaining and deepening meaningful social relationships. Thus, emotional engagement not only directly promotes well-being but also indirectly advances positive aging by strengthening social connectedness. (Reviewer2-3)Building upon this foundation, this study further posits that emotional engagement and social connectedness may form a chain mediation mechanism in the process through which digital micro-dramas influence active aging. Specifically, emotional engagement not only directly enhances elderly individuals’ subjective well-being and life satisfaction but also promotes active aging by strengthening social connectedness (Hypothesis H6). Based on the aforementioned theoretical framework and empirical evidence, this study hypothesizes that emotional engagement and social connectedness may jointly act to exert a chain mediation effect. By promoting social connectedness, emotional engagement indirectly enhances the level of active aging. Therefore, the following hypothesis is proposed:

Hypothesis 7a (H7a): Emotional engagement mediates the relationship between the narrativity of digital micro-dramas and active aging in elderly individuals.

Hypothesis 7b (H7b): Emotional engagement mediates the relationship between the emotional resonance of digital micro-dramas and active aging in elderly individuals.

Hypothesis 7c (H7c): Emotional engagement mediates the relationship between the interactivity of digital micro-dramas and active aging in elderly individuals.

Hypothesis 7d (H7d): Social connectedness mediates the relationship between the narrativity of digital micro-dramas and active aging in elderly individuals.

Hypothesis 7e (H7e): Social connectedness mediates the relationship between the emotional resonance of digital micro-dramas and active aging in elderly individuals.

Hypothesis 7f (H7f): Social connectedness mediates the relationship between the interactivity of digital micro-dramas and active aging in elderly individuals.

Hypothesis 7g (H7g): Emotional engagement and social connectedness jointly mediate the relationship between the narrativity of digital micro-dramas and active aging in elderly individuals.

Hypothesis 7h (H7h): Emotional engagement and social connectedness jointly mediate the relationship between the emotional resonance of digital micro-dramas and active aging in elderly individuals.

Hypothesis 7i (H7i): Emotional engagement and social connectedness jointly mediate the relationship between the interactivity of digital micro-dramas and active aging in elderly individuals.

### 2.2 Research model

**Figure 1:**
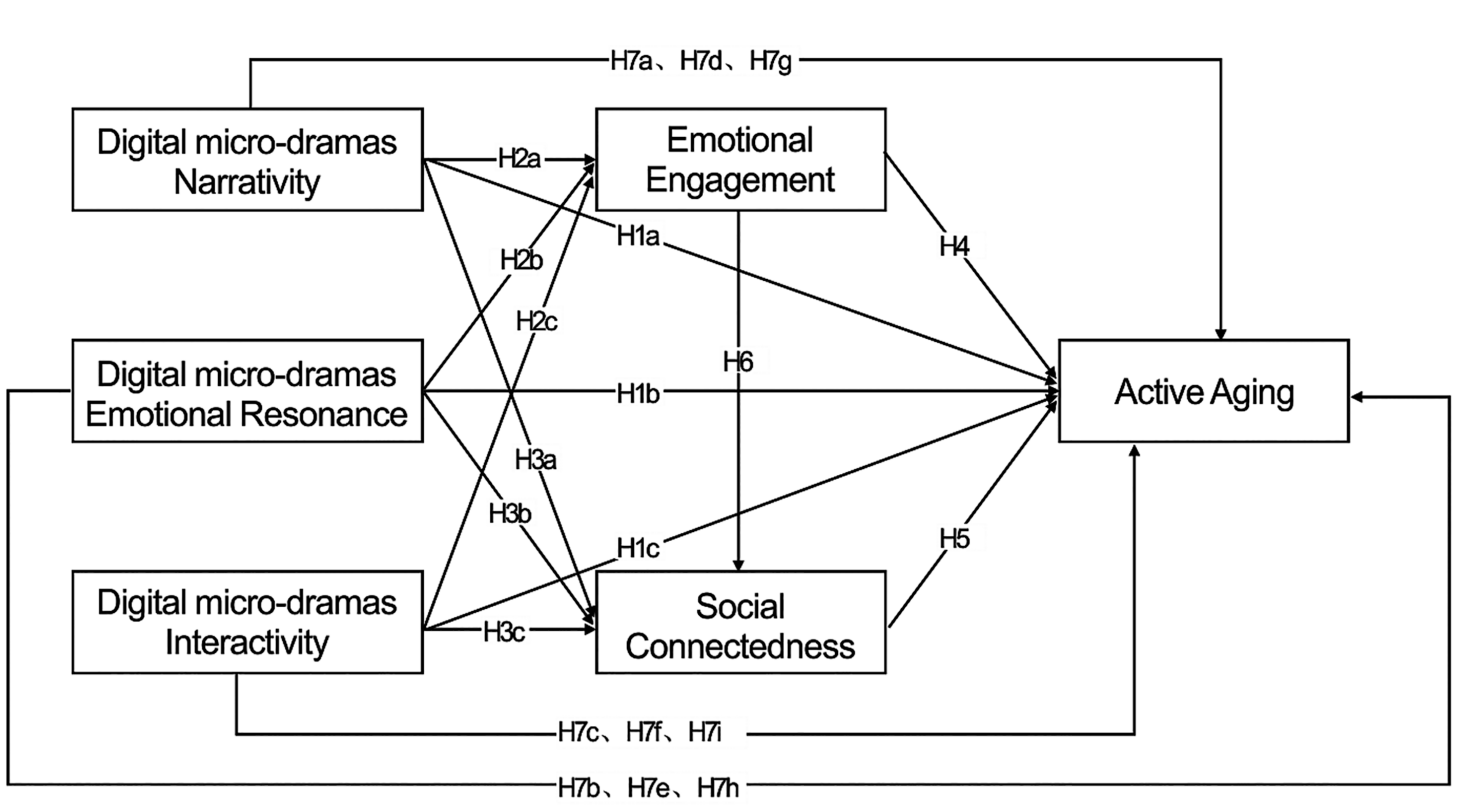
Model of the impact of digital micro-drama content characteristics on active aging.

This study’s conceptual model integrates the emotion–society mechanism with the positive aging theoretical framework. As shown in Figure 1, narrative quality, emotional resonance, and interactivity are regarded as the primary content characteristics influencing older audiences’ psychological responses. Narrative quality provides cognitive pathways, promoting immersion and meaning construction; emotional resonance elicits empathy and emotional arousal; and interactivity enhances participatory experiences through real-time feedback and social presence. Emotional engagement serves as the first-stage mediating variable, representing the internal psychological mechanism through which media experiences influence emotional states. Social connectedness functions as the second-stage mediating variable, reflecting the transformation of emotional experiences into social integration and active participation. This hierarchical structure aligns with emotion regulation theory (Gross, 2002) and social connection theory (Lee and Robbins, 1995), which posit that emotional engagement precedes and facilitates social bonding. Consequently, the constructed model reflects the dynamic psychosocial mechanisms through which digital media experiences influence positive aging. (Reviewer2-2).

## 3 Methodology

### 3.1 Data collection

This study developed the final questionnaire based on literature analysis, open-ended surveys, and multiple interviews. To ensure the questionnaire items were easy to understand, a pre-test was conducted with 25 elderly participants before the official survey. The official survey was designed and distributed using the professional online platform Wenjuanxing (wjx.cn), employing a random sampling method to survey individuals who met the target criteria. The participants were limited to elderly individuals aged 60 and above from mainland China who had previously watched digital micro-dramas.

Data were collected from June to July 2025, with a total of 750 questionnaires distributed and 750 returned. After data cleaning, 45 invalid questionnaires were excluded based on the following criteria: unusually short completion time, significant missing responses or repeated answers, logical contradictions in response options (e.g., all questions in the same scale answered identically), and responses that appeared unrealistic or randomly answered. A total of 705 valid questionnaires were retained. According to the sample size guidelines proposed by Kline (the required sample size should be at least 10 times the number of measurement items) (Kline, 2023), this study’s questionnaire utilized 24 measurement items, which means the required sample size should be at least 240. The actual valid sample size of 705 exceeds this minimum requirement, fully satisfying the needs for subsequent data analysis.

Notably, all participants in this study were seniors aged 60 and above who had previously watched digital micro-shorts; the sample did not include non-viewers. This research design aimed to ensure respondents possessed a fundamental understanding of the studied media format, enabling more accurate evaluations of its content characteristics and psychological effects. Among the initial 750 participants, approximately 81% of respondents indicated that they watched digital micro-shorts at least once per week, demonstrating high usage frequency and engagement with this medium among the elderly population. This finding corroborates the recent trend of active participation by older adults in consuming digital short-form content, particularly when such content carries strong emotional and social resonance (Chopik, 2016; Nimrod, 2020a, 2020b). Future research may consider including non-viewer groups in the sample to compare psychological and social outcomes across different levels of media exposure (Reviewer1-1).

### 3.2 Scale design

The six core variables involved in this study (narrativity, emotional resonance, interactivity, emotional engagement, social connectedness, and active aging) are all measured using established scales. As listed in Table 1, the scale items for each variable are primarily adapted from mature studies in the respective fields and have been revised and optimized to fit the specific context and target population of this research. All scale items use a 7-point Likert scale for measurement, where 1 represents “Strongly Disagree” and 7 represents “Strongly Agree” to assess the strength of the respondents’ attitudes.

**Table 1:** Questionnaire items and their sources.

Variables	Number	Items	Sources
Narrativity (NA)	NA1	These digital micro-dramas have a compact storyline with a clear structure.	Busselle and Bilandzic (2009); Cohen (2001); Green and Brock (2000)
	NA2	The story structure is coherent and complete, flowing naturally, which makes it easy for me to understand.	
	NA3	The story often features unexpected plot twists.	
	NA4	The beginning of the story is highly engaging, making me eager to continue watching.	
Emotional resonance (ER)	ER1	The beginning of the story is highly engaging, making me eager to continue watching.	Bilandzic et al. (2019); Davis (1983)
	ER2	These contents frequently bring me feelings of joy or pleasure.	
	ER3	I tend to empathize with the characters in the story (feeling nervous, happy, etc., for them).	
	ER4	After watching, I often find myself reflecting on the plot or thinking about its meaning.	
Interactivity (IN)	IN1	I can easily comment, like, send bullet comments, or interact through private messages.	Gomez-Uribe and Hunt (2016); Sundar and Limperos (2013)
	IN2	I use interactive features, such as branching story choices, autoplay, and speed adjustments.	
	IN3	I can easily use the platform’s sharing function to interact.	
	IN4	I participate in interactive activities on the platform’s community, such as storyline voting, discussions, and creating derivative content.	
Emotional engagement (EE)	EE1	While watching, I often immerse myself completely and care about the development of the plot.	Busselle and Bilandzic (2009); Green and Appel (2024)
	EE2	The storyline makes me experience emotional fluctuations (such as tension, anticipation, surprise).	
	EE3	I develop an emotional attachment to my favorite characters or scenes.	
	EE4	I look forward to the next episode or seek out similar micro-dramas to watch.	
Social connectedness (SC)	SC1	By watching micro-dramas, I have made new friends or expanded my social circle.	Derrick et al. (2009); Scheibe et al. (2022)
	SC2	I can receive support and responses from others in the comment section or group chats.	
	SC3	I have more topics to discuss with people who share similar interests, and we interact frequently.	
	SC4	My online interactions often extend to offline social activities.	
Active aging (AA)	AA1	Recently, I have become more proactive about health management and life goals.	Dong et al. (2023); Stargatt et al. (2022); Tsai et al. (2024)
	AA2	I enjoy participating in family, community, or online activities.	
	AA3	Lately, I am more willing to learn digital skills (such as mobile payments, video calls) to maintain social connectedness.	
	AA4	I am more motivated to make long-term life plans (such as travel, volunteer work).	

### 3.3 Data analysis

This study used SPSS 26 (IBM, USA) and SmartPLS 4 (SmartPLS GmbH, Germany) software for data processing and analysis. SmartPLS 4 was chosen primarily for its robust support for complex structural equation modeling (SEM), extensive modeling capabilities, and clear presentation of results. These features make it well-suited for testing the relationships between digital micro-drama content characteristics, emotional engagement, social connectedness, and active aging, as outlined in the research model.

The specific analysis process is as follows: first, descriptive statistics of the sample’s demographic characteristics, such as gender, age, and education level, were conducted using SPSS 26 to comprehensively understand the sample distribution. Next, the measurement model was evaluated using SmartPLS 4, testing the outer loadings, Cronbach’s Alpha, composite reliability (CR), and average variance extracted (AVE) to verify the reliability and validity of the scale. Finally, in the structural model evaluation and hypothesis testing phase, the Bootstrap sampling method in SmartPLS 4 was used to test the significance of path coefficients, and the model’s explanatory and predictive power was assessed using indicators such as R^2^ and Q^2^, thereby strengthening the empirical support for the relationships between variables and the theoretical hypotheses.

## 4 Results

### 4.1 Demographic characteristics of the respondents

Descriptive statistical analysis was conducted on 705 valid questionnaires, and the results are listed in Table 2. Notably, 52.62% of the respondents are male and 47.38% are female, with a relatively balanced gender ratio (MobTech, 2020). In terms of age distribution, the 60–65 age group accounts for 20.3%, whereas the 65–70 age group comprises 17%. These two age groups have higher proportions of respondents, reflecting that the sample is primarily composed of the elderly (Wu and Wen, 2023). Regarding education level, a large proportion of respondents have middle school or lower education, with 30.92% having completed middle school and 28.51% having primary school education. The percentage of respondents with higher education, such as college or university degrees, is relatively low, at 14.04 and 6.53%, respectively, indicating that the overall education level of the sample is on the lower side. Marital status shows a diverse distribution, with 22.27% being divorced, 20.99% married, 19.01% unmarried, and 16.88% widowed, reflecting the diversity in marital status within the elderly population. Regarding living arrangements, the majority of respondents live with relatives or friends (19.15%), followed by those living alone (16.74%), with children (16.03%), or with spouses (15.60%), showing the variety in living choices. Overall, the sample in this study is representative in terms of gender, age, education level, marital status, and living arrangements, providing a solid foundation for subsequent research.

**Table 2:** Demographic characteristics of respondents (sample size *n* = 705).

Category	Items	Frequency	Proportion
Gender	Female	334	47.376
	Male	371	52.624
Age (in years)	60–65	143	20.3
	65–70	120	17.0
	70–75	115	16.3
	75–80	125	17.7
	80–85	112	15.9
	>85	90	12.8
Education	Elementary School	201	28.511
	Middle School	218	30.922
	High School	123	17.447
	Junior College	99	14.043
	Undergraduate	46	6.525
	Graduate and Above	18	2.553
Marital status	Unmarried	134	19.007
	Married	148	20.993
	Divorced	157	22.270
	Widowed	119	16.879
	Other	147	20.851
Residential status	With spouse	110	15.603
	With children	113	16.028
	With other relatives and friends	135	19.149
	Institutional care	110	15.603
	Living alone	118	16.738
	Other	119	16.879

### 4.2 Reliability and validity analysis

A reliability analysis was conducted for the scales used in this survey, and the results indicated that all scales had high reliability. Specifically, as listed in Table 3, the standardized factor loadings for each item ranges from 0.68 to 0.76, which is significantly higher than the common threshold of 0.5. Moreover, it exceeds the ideal threshold of 0.7 (Cheung et al., 2024), which indicates the strong explanatory power of each item for its corresponding latent variable, providing strong support for the construct validity of the scale. The Cronbach’s α values ranged from 0.815 to 0.844, all exceeding the conventional reliability standard of 0.7 (Nunnally and Bernstein, 1994), indicating good internal consistency of the scale. CR values ranged from 0.817 to 0.845, which were all greater than the reference value of 0.6 (Bagozzi and Yi, 1988), and the average variance extracted (AVE) values ranged from 0.528 to 0.576, all exceeding the reference value of 0.5 (Hair et al., 2011). Notably, for some latent variables, CR was slightly lower than the α value, which could be due to the variation in item factor loadings. According to the recommendations of Fornell and Larcker (1981) and Hair et al. (2013), as long as CR > 0.6 and AVE > 0.5, the scale can be considered to have good convergent validity and reliability. Therefore, the results of this study are still reliable.

**Table 3:** Reliability and validity analysis.

Construct	Item	Factor loadings	Cronbach’s alpha	CR	AVE
Narrativity (NA)	NA1	0.732	0.825	0.819	0.530
	NA2	0.758			
	NA3	0.688			
	NA4	0.733			
Emotional resonance (ER)	ER1	0.757	0.838	0.842	0.571
	ER2	0.708			
	ER3	0.762			
	ER4	0.794			
Interactivity (IN)	IN1	0.754	0.842	0.839	0.565
	IN2	0.732			
	IN3	0.758			
	IN4	0.763			
Emotional engagement (EE)	EE1	0.749	0.818	0.827	0.544
	EE2	0.694			
	EE3	0.767			
	EE4	0.738			
Social connectedness (SC)	SC1	0.756	0.844	0.845	0.576
	SC2	0.773			
	SC3	0.775			
	SC4	0.732			
Active aging (AA)	AA1	0.772	0.815	0.817	0.528
	AA2	0.755			
	AA3	0.67			
	AA4	0.706			

### 4.3 Discriminant validity analysis

This study employs two methods to verify the discriminant validity of the model. The first method is the Fornell–Larcker criterion, which states that the correlation between latent variables should be smaller than the square root of their respective AVEs. The second method is the Heterotrait–Monotrait ratio (HTMT), with the criterion that the HTMT value should be below 0.85 (Henseler et al., 2015). The discriminant validity of the model in this study was verified by both methods. The results are listed in Tables 4, 5.

**Table 4:** Distinctive validity analysis (Fornell–Larcker).

	AA	EE	ER	IN	NA	SC
Active aging (AA)	0.727					
Emotional engagement (EE)	0.571	0.738				
Emotional resonance (ER)	0.550	0.483	0.756			
Interactivity (IN)	0.589	0.480	0.500	0.752		
Narrativity (NA)	0.553	0.508	0.509	0.485	0.728	
Social connectedness (SC)	0.562	0.505	0.471	0.466	0.579	0.759

**Table 5:** Discrimination validity analysis (HTML).

	AA	EE	ER	IN	NA	SC
Active aging (AA)						
Emotional engagement (EE)	0.579					
Emotional resonance (ER)	0.553	0.483				
Interactivity (IN)	0.594	0.487	0.499			
Narrativity (NA)	0.567	0.518	0.513	0.489		
Social connectedness (SC)	0.575	0.513	0.469	0.468	0.579	

### 4.4 Multicollinearity analysis

Multicollinearity diagnosis is the first step in assessing the results of the structural model. The criterion for success is that the variance inflation factor (VIF) of the internal model should be less than 5 (Hair et al., 2021), which indicates that there are no serious multicollinearity issues in the data. As listed in Table 6, the VIF values range from 1.469 to 1.720. Thus, the structural model constructed in this study passes the multicollinearity diagnosis.

**Table 6:** Collinearity diagnosis.

Items	VIF	Tolerance
Narrativity (NA)	1.557	0.642
Emotional resonance (ER)	1.469	0.681
Narrativity (IN)	1.495	0.669
Emotional engagement (EE)	1.496	0.668
Social connectedness (SC)	1.551	0.645
Active aging (AA)	1.720	0.581

### 4.5 Path analysis

**Figure 2:**
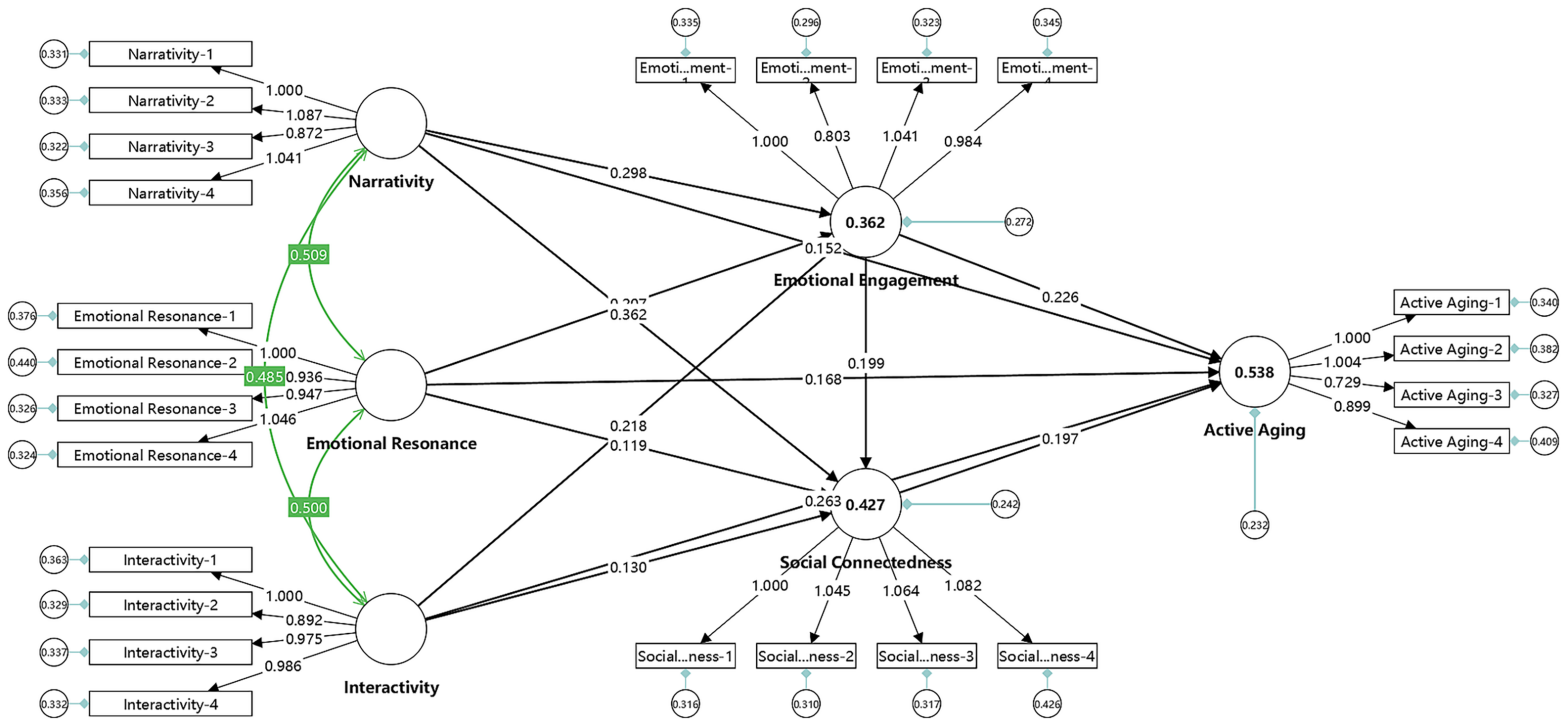
Model analysis results.

The path coefficients of the structural model were evaluated using SmartPLS4. When the t-value of the path coefficient is greater than 1.96 (Bagozzi and Yi, 1988), it indicates that the path coefficient passes the test at the 5% significance level, showing statistical significance. The analysis results are presented in Table 7 and Figure 2. The details are as follows:

**Table 7:** Path analysis.

Hypothesis	β	STDEV	T	P	Decision		
					2.5%	97.5%	Decision
H1a: NA → AA	0.133	0.050	2.666	0.008	0.034	0.230	Supported
H1b: ER → AA	0.168	0.046	3.625	0.000	0.077	0.256	Supported
H1c: IN → AA	0.257	0.049	5.225	0.000	0.160	0.352	Supported
H2a: NA → EE	0.282	0.053	5.366	0.000	0.182	0.384	Supported
H2b: ER → EE	0.225	0.052	4.316	0.000	0.121	0.321	Supported
H2c: IN → EE	0.231	0.048	4.758	0.000	0.137	0.326	Supported
H3a: NA → SC	0.344	0.049	6.958	0.000	0.249	0.443	Supported
H3b: ER → SC	0.130	0.049	2.633	0.009	0.033	0.224	Supported
H3c: IN → SC	0.138	0.049	2.798	0.005	0.041	0.232	Supported
H4: EE → AA	0.208	0.047	4.425	0.000	0.116	0.297	Supported
H5: SC → AA	0.181	0.051	3.523	0.000	0.084	0.284	Supported
H6: EE → SC	0.200	0.051	3.923	0.000	0.099	0.298	Supported
H7a: NA → EE → AA	0.059	0.018	3.313	0.001	0.028	0.098	Supported
H7b: ER → EE → AA	0.047	0.016	3.016	0.003	0.020	0.081	Supported
H7c: IN → EE → AA	0.048	0.015	3.231	0.001	0.022	0.080	Supported
H7d: NA → SC → AA	0.062	0.02	3.081	0.002	0.027	0.106	Supported
H7e: ER → SC → AA	0.024	0.012	2.006	0.045	0.006	0.052	Supported
H7f: IN → SC → AA	0.025	0.012	2.118	0.034	0.008	0.056	Supported
H7g: NA → EE → SC → AA	0.01	0.004	2.484	0.013	0.004	0.022	Supported
H7h: ER → EE → SC → AA	0.008	0.004	2.181	0.029	0.003	0.018	Supported
H7i: IN → EE → SC → AA	0.008	0.004	2.235	0.026	0.003	0.019	Supported

NA (β = 0.133, T = 2.666, *p* = 0.008), ER (β = 0.168, T = 3.625, *p* = 0.000), and IN (β = 0.257, T = 5.225, p = 0.000) have a significant positive effect on AA. Therefore, hypotheses H1a, H1b, and H1c are supported.

NA (β = 0.282, T = 5.366, p = 0.000), ER (β = 0.225, T = 4.316, p = 0.000), and IN (β = 0.231, T = 4.757, p = 0.000) have a significant positive effect on EE. Therefore, hypotheses H2a, H2b, and H2c are supported.

NA (β = 0.344, T = 6.958, p = 0.000), ER (β = 0.130, T = 2.633, *p* = 0.009), and IN (β = 0.138, T = 2.798, *p* = 0.005) have a significant positive effect on SC. Therefore, hypotheses H3a, H3b, and H3c are supported.

EE (β = 0.208, T = 4.425, p = 0.000) has a significant positive effect on AA. Therefore, hypothesis H4 is supported.

SC (β = 0.181, T = 3.523, p = 0.000) has a significant positive effect on AA. Therefore, hypothesis H5 is supported.

EE (β = 0.200, T = 3.923, p = 0.000) has a significant positive effect on SC. Therefore, hypothesis H6 is supported.

In addition, EE mediates the relationship between NA and AA (β = 0.059, T = 3.313, *p* = 0.001), ER and AA (β = 0.047, T = 3.016, *p* = 0.003), and IN and AA (β = 0.048, T = 3.231, p = 0.001). Therefore, hypotheses H7a, H7b, and H7c are supported.

SC mediates the relationship between NA and AA (β = 0.062, T = 3.081, *p* = 0.002), ER and AA (β = 0.024, T = 2.006, *p* = 0.045), and IN and AA (β = 0.025, T = 2.118, *p* = 0.034). Therefore, hypotheses H7d, H7e, and H7f are supported.

EE and SC exhibit a serial mediation effect between NA and AA (β = 0.010, T = 2.484, *p* = 0.013), ER and AA (β = 0.008, T = 2.181, *p* = 0.029), and IN and AA (β = 0.008, T = 2.235, *p* = 0.026). Therefore, hypotheses H7g, H7h, and H7i are supported.

### 4.6 Model explanatory power and predictive ability

In this study, covariance-based structural equation modeling (CB-SEM) was used to analyze the causal relationships between latent variables and assess the model’s explanatory power and predictive ability. The explanatory power of a model is primarily measured by the R^2^ value of endogenous variables, which reflects the proportion of variance in the endogenous variables explained by the exogenous variables. When the R^2^ value is greater than 0.25 (Sarstedt et al., 2014), it indicates that the model has a strong explanatory power for the endogenous variables. To further evaluate the predictive ability of the model, the Stone–Geisser Q^2^ test under the PLS-SEM framework was applied. If Q^2^ > 0, the model has predictive relevance for the endogenous variables (Hair Jr et al., 2018). As listed in Table 8, the model in this study not only demonstrates good explanatory power but also exhibits robust predictive ability, providing a reliable foundation for the analysis of causal relationships between latent variables.

**Table 8:** R^2^ value and Q^2^ value.

	R^2^	Q^2^
Active aging	0.538	0.357
Emotional engagement	0.362	0.272
Social connectedness	0.427	0.300

## 5 Discussion and implications

### 5.1 Discussion

Existing research on active aging has focused on the roles of physical exercise, community participation, or traditional media (such as television)(Nimrod, 2020a; Silverstein and Parker, 2002), but the impact of emerging digital cultural forms, especially micro-dramas, on the psychosocial functions of the elderly has not been sufficiently studied. The empirical results of this study demonstrate that the narrative, emotional resonance, and interactivity of digital micro-dramas can significantly promote active aging. Moreover, the mediating and serial mediating roles of emotional engagement and social connectedness in this process are revealed. This enriches the theory of active aging and expands the applicability of research on digital media.

First, the narrative, emotional resonance, and interactivity of digital micro-dramas all have a significant positive impact on active aging. This is consistent with existing research on the positive effects of media narratives and emotional engagement on the mental health and social integration of the elderly (Cohen and Wills, 1985; Green and Brock, 2000). Unlike previous studies that focused on long films, literary narratives, or television programs, this study validates the value of short-duration, digital narratives in promoting active aging. The data show that interactivity has the greatest impact on active aging, indicating that elderly individuals are more likely to gain social support and a sense of self-worth through actions such as liking, commenting, and sharing, thereby achieving psychological satisfaction and social integration in the digital environment.

Furthermore, the results demonstrate that the narrative, emotional resonance, and interactivity of digital micro-dramas significantly enhance emotional engagement, which aligns with existing research, identifying emotional responses as the core psychological mechanism of media narratives (Nabi and Green, 2014). Among these factors, the role of narrative is the most prominent, indicating that elderly audiences are more likely to experience immersion through plot development and character building, thereby stimulating emotional involvement and psychological resonance (Green and Brock, 2000). This suggests that the concise and focused narrative format of micro-dramas can quickly evoke emotional responses from the elderly, enhancing media engagement. In other words, narrative provides the core driving force, whereas emotional resonance and interactivity further strengthen it, collectively shaping a positive digital emotional experience.

In addition, the study highlights that the narrative, emotional resonance, and interactivity of micro-dramas significantly enhance social connectedness. This is consistent with the research, which emphasizes that emotional experience and media interaction promote group belongingness (Cohen and Wills, 1985). The data reveal that the narrative aspect of digital micro-dramas has the strongest effect on social connectedness, indicating that elderly audiences are more likely to establish a sense of identification and shared memory through the storyline and character relationships, thereby narrowing the psychological distance to others and strengthening social bonds. This result validates the social identity theory’s hypothesis that shared narratives foster group cohesion and demonstrates that micro-dramas, through their concise narrative form, can effectively and quickly stimulate intergenerational communication and social interaction. In other words, narrative lays the foundation for social connectedness, whereas emotional resonance and interactivity play a complementary and expansive role, collectively promoting social participation and a sense of belonging for the elderly in the digital environment.

Moreover, emotional engagement and social connectedness both have a significant positive impact on active aging. This result is highly consistent with research showing that positive emotions and social support promote well-being (Fredrickson, 2001; Litwin and Shiovitz-Ezra, 2011). Previous studies have focused on emotions or social factors separately, but this study reveals their synergistic effect in the digital environment. Emotional engagement can enhance psychological capital, thus helping elderly individuals adapt to aging (Carstensen et al., 2003), whereas social connectedness significantly alleviates feelings of loneliness and isolation by strengthening support networks (Haslam et al., 2018). This result indicates that active aging depends not only on individual emotional experiences but also on the expansion and maintenance of social relationships. These two aspects complement each other and together form the key mechanism that drives active aging among the elderly in the digital media environment.

Further analysis shows that emotional engagement significantly promotes social connectedness, which aligns with SST, emphasizing the important role of emotional experiences in choosing and maintaining interpersonal relationships (Carstensen et al., 1999). Positive emotions enhance empathy and willingness to share, thereby strengthening group identity and belongingness (Diener and Seligman, 2002). In the context of micro-dramas, elderly individuals convert personal emotions into social interactions through comments, shares, and other forms of engagement, thus enhancing intergenerational communication and cohesion (Jenkins, 2006). This result reveals the interactive effect of the emotional–social mechanism.

Finally, this study elucidates mediating and serial mediating mechanisms. Emotional engagement plays a partial mediating role in the relationship between narrative, emotional resonance, and interactivity and active aging, which aligns with the explanation of the core function of emotional experience in emotion regulation theory (Gross, 2002). Social connectedness also plays a mediating role, indicating that micro-dramas promote elderly individuals’ life satisfaction and mental health by enhancing social interaction and a sense of support (Putnam, 2000). More importantly, the serial mediation effect shows that emotional engagement indirectly promotes active aging through social connectedness, indicating that digital micro-dramas not only affect individual emotions but also exert a more systematic influence on active aging through social networks.

Notably, although all mediation paths are significantly supported, the differences in their effect sizes also reveal the hierarchical nature of the mechanisms. For example, the indirect effect of emotional engagement on active aging is stronger overall than the indirect effect through social connectedness, indicating that emotional experience remains the core driving force of elderly individuals’ active media participation and psychological transformation. Social connectedness, on the other hand, plays a complementary and extending role, forming an emotional–social serial chain. This difference not only reflects the importance ranking of different psychological and social mechanisms but also suggests that practice should prioritize triggering elderly people’s positive media experiences by strengthening emotional resonance and interactive design, followed by consolidation through community-based social mechanisms.

Overall, this study expands the application of active aging theory in the context of digital culture, which is of academic and practical value. The findings suggest that digital micro-dramas can promote active aging by stimulating emotional engagement and enhancing social connectedness, thus providing theoretical support for digital content design. Second, this study reveals the dual role pathways of psychological and social mechanisms, elucidating the psychological processes behind elderly media usage. Finally, the findings provide practical guidance for digital content creators, elderly service institutions, and public cultural managers, helping them to enhance the psychological well-being and social participation levels of the elderly population.

### 5.2 Implications

This study developed an analytical model of digital micro-drama content characteristics–emotional engagement–social connectedness–active aging and empirically tested its mechanism. In so doing, it fills a gap in existing research on active aging that has overlooked emerging digital media. The results revealed that the narrative, emotional resonance, and interactivity of micro-dramas significantly enhance the emotional engagement and social connectedness of elderly audiences, thereby promoting active aging. This finding not only expands the application of the emotional–social mechanism model in the context of digital culture but also enriches the theoretical framework of media psychology and social gerontology, providing new evidence for understanding the role of short-duration, highly interactive digital content in the psychological and social adaptation of elderly groups.

At the practical level, the research results provide actionable references for digital content creators, elderly care service institutions, and public cultural managers. Digital micro-dramas can enhance elderly individuals’ emotional involvement and sense of social belonging through immersive storytelling and interactive experiences, and the results offer empirical guidance for designing digital cultural products targeted at the elderly. Meanwhile, the study suggests that public cultural policies and active aging strategies should focus on the psychosocial effects of digital media, integrating them into pathways for increasing elderly social participation, intergenerational communication, and psychological well-being. Overall, this study not only provides a new perspective for theoretical development but also offers practical strategies that can be adopted, contributing to the integration of digital culture with aging society governance.

## 6 Conclusion, limitations, and future research

This study explores the impact of the content characteristics of digital micro-dramas on active aging and conducts a theoretical and empirical analysis through the chain mediation model of emotional engagement and social connectedness. The results show that the narrative, emotional resonance, and interactivity of micro-dramas not only enhance emotional engagement among the elderly but also play an important role in strengthening social connectedness, thereby collectively improving active aging levels. This finding not only expands the applicability of media psychology in elderly populations but also provides new evidence for active aging theory in the context of digital culture.

However, this study has certain limitations. First, the research uses a cross-sectional survey method, which makes it difficult to reveal the dynamic changes in causal relationships. Elderly individuals’ preferences for digital micro-dramas and their psychological effects may change over time, but this study was unable to fully capture this process. Second, the sample mainly comes from urban areas and has a certain level of cultural and educational representativeness, which may limit the external validity of the study results. For elderly individuals in rural areas or groups with limited digital access, the acceptance and mechanisms of micro-dramas may differ. Additionally, this study primarily relies on self-reported questionnaires, and although social desirability bias has been controlled for, overestimation or underestimation by respondents may still be present in terms of emotional experience and social connectedness.

Future research could expand in the following directions. First, longitudinal tracking or experimental methods should be employed to further verify the long-term effects and causal mechanisms of digital micro-dramas on active aging. Second, a cross-cultural comparison could be made to examine the acceptance and influence paths of micro-dramas among elderly individuals in different social environments to test the generalizability of the research conclusions. Third, future studies could combine qualitative research methods to explore in greater detail how elderly individuals experience micro-drama content through narrative, emotional resonance, and interactivity, revealing more intricate psychological processes and social effects. Finally, with the development of technologies such as artificial intelligence and virtual reality, future research could also focus on the potential role of immersive media content in regulating emotions, enhancing social connectedness, and promoting active aging among elderly groups, thus providing more forward-looking theoretical and practical support for the integration of digital culture and healthy aging.

## Data Availability

The original contributions presented in the study are included in the article/supplementary material, further inquiries can be directed to the corresponding author.

## References

[ref1] Ajith T. N P. S Mathew L. S. (2025). Experience matters: exploring the impact of user experience on stickiness and loyalty in OTT platforms. Int. J. Hum.-Comput. Interact. 41, 11363–11377. doi: 10.1080/10447318.2024.2443245

[ref2] Bagozzi R. P. Yi Y. (1988). On the evaluation of structural equation models. J. Acad. Mark. Sci. 16, 74–94. doi: 10.1007/BF02723327

[ref3] Barbosa Neves B. Franz R. Judges R. Beermann C. Baecker R. (2017). Can digital technology enhance social connectedness among older adults? A feasibility study. J. Appl. Gerontol. 38, 49–72. doi: 10.1177/0733464817741369, PMID: 29166818

[ref4] Barsade S. G. (2002). The ripple effect: emotional contagion and its influence on group behavior. Adm. Sci. Q. 47, 644–675. doi: 10.2307/3094912

[ref5] Berger J. Milkman K. L. (2012). What makes online content viral? J. Mark. Res. 49, 192–205. doi: 10.1509/jmr.10.0353

[ref6] Berkman L. F. Glass T. Brissette I. Seeman T. E. (2000). From social integration to health: Durkheim in the new millennium. Soc. Sci. Med. 51, 843–857. doi: 10.1016/S0277-9536(00)00065-4, PMID: 10972429

[ref7] Bilandzic H. Sukalla F. Schnell C. Hastall M. R. Busselle R. W. (2019). The narrative engageability scale: a multidimensional trait measure for the propensity to become engaged in a story. Int. J. Commun. 13, 801–832.

[ref8] Bohlmeijer E. Roemer M. Cuijpers P. Smit F. (2007). The effects of reminiscence on psychological well-being in older adults: a meta-analysis. Aging Ment. Health 11, 291–300. doi: 10.1080/13607860600963547, PMID: 17558580

[ref9] Bowling A. (2008). Enhancing later life: how older people perceive active ageing? Aging Ment. Health 12, 293–301. doi: 10.1080/13607860802120979, PMID: 18728941

[ref10] Busselle R. Bilandzic H. (2009). Measuring narrative engagement. Media Psychol. 12, 321–347. doi: 10.1080/15213260903287259

[ref11] Carstensen L. L. (1992). Social and emotional patterns in adulthood: support for socioemotional selectivity theory. Psychol. Aging 7, 331–338. doi: 10.1037/0882-7974.7.3.331, PMID: 1388852

[ref12] Carstensen L. L. (2006). The influence of a sense of time on human development. Science 312, 1913–1915. doi: 10.1126/science.1127488, PMID: 16809530 PMC2790864

[ref13] Carstensen L. L. Fung H. H. Charles S. T. (2003). Socioemotional selectivity theory and the regulation of emotion in the second half of life. Motiv. Emot. 27, 103–123. doi: 10.1023/A:1024569803230

[ref14] Carstensen L. L. Isaacowitz D. M. Charles S. T. (1999). Taking time seriously: a theory of socioemotional selectivity. Am. Psychol. 54, 165–181. doi: 10.1037/0003-066X.54.3.165, PMID: 10199217

[ref15] Carstensen L. L. Mikels J. A. Mather M. (2006). “Aging and the intersection of cognition, motivation, and emotion” in Handbook of the psychology of aging. eds. Birren J. Schaie K. W.. 6th ed, 343–362. doi: 10.1016/B978-012101264-9/50018-5

[ref16] Chai Y. Xian G. Wang M. Guo L. Luo S. (2024). Aging wisely: the impact of internet use on older adults' mental health. J. Affect. Disord. 364, 139–145. doi: 10.1016/j.jad.2024.08.076, PMID: 39147146

[ref17] Chen J. (2024). A study on the influencing factors of older adults’ use of short play under the “digital feedback” Perspective. doi: 10.2139/ssrn.4705382

[ref18] Chen C. Huang N. Hu B. Zhang M. Yuan J. Guo J. (2025). The effectiveness of digital technology interventions for cognitive function in older adults: a systematic review and meta-analysis of randomized controlled trials. GeroScience 47, 653–683. doi: 10.1007/s11357-024-01446-z, PMID: 39688787 PMC11872853

[ref19] Chen Y. R. Schulz P. J. (2016). The effect of ICT interventions on reducing social isolation in the elderly: a systematic review. J. Med. Internet Res. 18:e18. doi: 10.2196/jmir.4596, PMID: 26822073 PMC4751336

[ref20] Chen B. Yang C. Ren S. Li P. Zhao J. (2024). Relationship between internet use and cognitive function among middle-aged and older Chinese adults: 5-year longitudinal study. J. Med. Internet Res. 26:e57301. doi: 10.2196/57301, PMID: 39539034 PMC11660964

[ref21] Chen L. Zhang Z. (2022). Community participation and subjective well-being of older adults: the roles of sense of community and neuroticism. Int. J. Environ. Res. Public Health 19:3261. doi: 10.3390/ijerph19063261, PMID: 35328950 PMC8953512

[ref22] Cheong J. H. Molani Z. Sadhukha S. Chang L. J. (2023). Synchronized affect in shared experiences strengthens social connection. Commun. Biol. 6:1099. doi: 10.1038/s42003-023-05461-2, PMID: 37898664 PMC10613250

[ref23] Cheung G. W. Cooper-Thomas H. D. Lau R. S. Wang L. C. (2024). Reporting reliability, convergent and discriminant validity with structural equation modeling: a review and best-practice recommendations. Asia Pac. J. Manag. 41, 745–783. doi: 10.1007/s10490-023-09871-y

[ref24] Choi M. Kong S. Jung D. (2012). Computer and internet interventions for loneliness and depression in older adults: a meta-analysis. Healthcare Inform. Res. 18, 191–198. doi: 10.4258/hir.2012.18.3.191, PMID: 23115742 PMC3483477

[ref25] Chopik W. J. (2016). The benefits of social technology use among older adults are mediated by reduced loneliness. Cyberpsychol. Behav. Soc. Netw. 19, 551–556. doi: 10.1089/cyber.2016.0151, PMID: 27541746 PMC5312603

[ref26] Cohen J. (2001). Defining identification: a theoretical look at the identification of audiences with media characters. Mass Commun. Soc. 4, 245–264. doi: 10.1207/S15327825MCS0403_01

[ref27] Cohen S. Wills T. A. (1985). Stress, social support, and the buffering hypothesis. Psychol. Bull. 98, 310–357. doi: 10.1037/0033-2909.98.2.310, PMID: 3901065

[ref28] Cotten S. R. Anderson W. A. McCullough B. M. (2013). Impact of internet use on loneliness and contact with others among older adults. Comput. Hum. Behav. 29, 140–149. doi: 10.2196/jmir.2306 PMC3636305 23448864

[ref29] Cotten S. R. Schuster A. M. Seifert A. (2022). Social media use and well-being among older adults. Curr. Opin. Psychol. 45:101293. doi: 10.1016/j.copsyc.2021.12.005, PMID: 35065352

[ref30] Cruwys T. Dingle G. A. Haslam C. Haslam S. A. Jetten J. Morton T. A. (2013). Social group memberships protect against future depression, alleviate depression symptoms and prevent depression relapse. Soc. Sci. Med. 98, 179–186. doi: 10.1016/j.socscimed.2013.09.013, PMID: 24331897

[ref31] Cui K. Zou W. Ji X. (2024). Does digital technology make people healthier: the impact of digital use on the lifestyle of Chinese older adults. BMC Geriatr. 24:85. doi: 10.1186/s12877-023-04651-1, PMID: 38254001 PMC10804579

[ref32] Davis H. M. (1983). Measuring individual differences in empathy: evidence for a multidimensional approach. J. Pers. Soc. Psychol. 44, 113–126. doi: 10.1037/0022-3514.44.1.113

[ref33] Decety J. Jackson P. L. (2004). The functional architecture of human empathy. Behav. Cogn. Neurosci. Rev. 3, 71–100. doi: 10.1177/1534582304267187, PMID: 15537986

[ref34] Derrick J. L. Gabriel S. Hugenberg K. (2009). Social surrogacy: how favored television programs provide the experience of belonging. J. Exp. Soc. Psychol. 45, 352–362. doi: 10.1016/j.jesp.2008.12.003

[ref35] Diener E. Seligman M. E. P. (2002). Very happy people. Psychol. Sci. 13, 81–84. doi: 10.1111/1467-9280.00415, PMID: 11894851

[ref36] Dong Q. Liu T. Liu R. Yang H. Liu C. (2023). Effectiveness of digital health literacy interventions in older adults: single-arm meta-analysis. J. Med. Internet Res. 25:e48166. doi: 10.2196/48166, PMID: 37379077 PMC10365623

[ref37] Ellison N. B. Steinfield C. Lampe C. (2007). The benefits of Facebook “friends:” social capital and college students’ use of online social network sites. J. Comput.-Mediat. Commun. 12, 1143–1168. doi: 10.1111/j.1083-6101.2007.00367.x

[ref38] Eyal K. Cohen J. (2006). When good friends say goodbye: a parasocial breakup study. J. Broadcast. Electron. Media 50, 502–523. doi: 10.1207/s15506878jobem5003_9

[ref39] Fornell C. Larcker D. F. (1981). Evaluating structural equation models with unobservable variables and measurement error. J. Mark. Res. 18, 39–50. doi: 10.1177/002224378101800104

[ref40] Fredrickson B. L. (2001). The role of positive emotions in positive psychology: the broaden-and-build theory of positive emotions. Am. Psychol. 56, 218–226. doi: 10.1037/0003-066X.56.3.218, PMID: 11315248 PMC3122271

[ref41] Fredrickson B. L. (2004). The broaden-and-build theory of positive emotions. Philos. Trans. R. Soc. Lond. Ser. B Biol. Sci. 359, 1367–1377. doi: 10.1098/rstb.2004.1512, PMID: 15347528 PMC1693418

[ref42] Gao Y. Liang J. Xu Z. (2024). Digital social media expression and social adaptability of older adults driven by artificial intelligence. Front. Public Health 12:1424898. doi: 10.3389/fpubh.2024.1424898, PMID: 39267635 PMC11390374

[ref43] Garcia D. Kappas A. Küster D. Schweitzer F. (2016). The dynamics of emotions in online interaction. R. Soc. Open Sci. 3:59. doi: 10.1098/rsos.160059, PMID: 27853586 PMC5108936

[ref44] Gerrig R. J. (1993). Experiencing narrative worlds: On the psychological activities of reading: Yale University Press. Avilable online at: http://www.jstor.org/stable/j.ctt1dr37cb

[ref45] Gomez-Uribe C. A. Hunt N. (2016). The Netflix recommender system: algorithms, business value, and innovation. ACM Trans. Manag. Inf. Syst. 6:13. doi: 10.1145/2843948

[ref46] Green M. C. Appel M. (2024). Narrative transportation: how stories shape how we see ourselves and the world. Adv. Exp. Soc. Psychol. 70, 1–82. doi: 10.1016/bs.aesp.2024.03.002, PMID: 41136281

[ref47] Green M. C. Brock T. C. (2000). The role of transportation in the persuasiveness of public narratives. J. Pers. Soc. Psychol. 79, 701–721. doi: 10.1037//0022-3514.79.5.701, PMID: 11079236

[ref48] Green M. C. Brock T. C. Kaufman G. F. (2004). Understanding media enjoyment: the role of transportation into narrative worlds. Commun. Theory 14, 311–327. doi: 10.1111/j.1468-2885.2004.tb00317.x

[ref49] Gross J. J. (2002). Emotion regulation: affective, cognitive, and social consequences. Psychophysiology 39, 281–291. doi: 10.1017/S0048577201393198, PMID: 12212647

[ref50] Hair J. F. Black W. C. Babin B. J. Anderson R. E. Tatham R. L. (2013). Multivariate Data Analysis. Edinburgh Gate, Harlow: Pearson Education Limited.

[ref51] Hair J. F. Jr. Hult G. T. M. Ringle C. M. Sarstedt M. Danks N. P. Ray S. (2021). Partial least squares structural equation modeling (PLS-SEM) using R: A workbook: Springer Nature.

[ref52] Hair J. F. Sarstedt M. Ringle C. M. Gudergan S. P. (2018). Advanced Issues in Partial Least Squares Structural Equation Modeling (PLS-SEM). Thousand Oaks, CA: Sage. doi: 10.3926/oss.37

[ref53] Hair J. F. Ringle C. M. Sarstedt M. (2011). PLS-SEM: indeed a silver bullet. J. Mark. Theory Pract. 19, 139–152. doi: 10.2753/MTP1069-6679190202

[ref54] Hamilton W. A. Garretson O. Kerne A. (2014). “Streaming on twitch: fostering participatory communities of play.” In Proceedings of the SIGCHI Conference on Human Factors in Computing Systems,

[ref55] Haslam C. Jetten J. Cruwys T. Dingle G. A. Haslam S. A. (2018). The new psychology of health: Unlocking the social cure, Routledge.

[ref56] Hatfield E. Cacioppo J. T. Rapson R. L. (1993). Emotional contagion. Curr. Dir. Psychol. Sci. 2, 96–100. doi: 10.1111/1467-8721.ep10770953

[ref57] Havighurst R. J. (1961). Successful aging. Gerontologist 1, 8–13. doi: 10.1093/geront/1.1.8

[ref58] Henseler J. Ringle C. M. Sarstedt M. (2015). A new criterion for assessing discriminant validity in variance-based structural equation modeling. J. Acad. Mark. Sci. 43, 115–135. doi: 10.1007/s11747-014-0403-8

[ref59] Heo J. Chun S. Lee S. Lee K. H. Kim J. (2015). Internet use and well-being in older adults. Cyberpsychol. Behav. Soc. Netw. 18, 268–272. doi: 10.1089/cyber.2014.0549, PMID: 25919967

[ref60] Higgins T. (2023). China tried to keep kids off social media. Now the elderly are hooked. Wired.

[ref61] Hilvert-Bruce Z. Neill J. T. Sjöblom M. Hamari J. (2018). Motivations of live-streaming viewer engagement. Comput. Hum. Behav. 84, 58–67. doi: 10.1016/j.chb.2018.02.013

[ref62] Hollebeek L. D. Glynn M. S. Brodie R. J. (2014). Consumer brand engagement in social media: conceptualization, scale development and validation. J. Interact. Mark. 28, 149–165. doi: 10.1016/j.intmar.2013.12.002

[ref63] Holt-Lunstad J. (2022). Social connection as a public health issue: the evidence and a systemic framework for prioritizing the "social" in social determinants of health. Annu. Rev. Public Health 43, 193–213. doi: 10.1146/annurev-publhealth-052020-110732, PMID: 35021021

[ref64] Holt-Lunstad J. Smith T. B. Layton J. B. (2010). Social relationships and mortality risk: a meta-analytic review. PLoS Med. 7:e1000316. doi: 10.1371/journal.pmed.1000316, PMID: 20668659 PMC2910600

[ref65] House J. S. Landis K. R. Umberson D. (1988). Social relationships and health. Science 241, 540–545. doi: 10.1126/science.3399889, PMID: 3399889

[ref66] Huang Y. Sun Y. (2011). “Emotion-driven interactive digital storytelling” in Lecture notes in computer science. eds. Zhou M. Li H. Deng R., vol. 6972 (Springer), 22–27.

[ref67] Hughes M. E. Waite L. J. Hawkley L. C. Cacioppo J. T. (2008). A short scale for measuring loneliness in large surveys: results from two population-based studies. Res. Aging 30, 619–638. doi: 10.1177/0164027504268574 PMC2394670 18504506

[ref68] Igartua J. J. Vega Casanova J. (2016). Identification with characters, elaboration, and Counterarguing in entertainment-education interventions through audiovisual fiction. J. Health Commun. 21, 293–300. doi: 10.1080/10810730.2015.1064494, PMID: 26166213

[ref69] Iqbal S. Fischl C. Asai R. (2025). Older persons’ social participation, health and well-being through digital engagement. Act. Adapt. Aging 1-30, 1–30. doi: 10.1080/01924788.2025.2512299, PMID: 40989069

[ref70] Jenkins H. (2006). Convergence culture: Where old and new media collide. New York, NY: New York University Press.

[ref71] Johnson D. R. (2012). Transportation into a story increases empathy, prosocial behavior, and perceptual bias toward fearful expressions. Pers. Individ. Differ. 52, 150–155. doi: 10.1016/j.paid.2011.10.005

[ref72] Junxun C. (2024). A study on the influencing factors of older adults' use of Short play under the “digital feedback” perspective.

[ref73] Kang J. A. Hong S. Hubbard G. T. (2020). The role of storytelling in advertising: consumer emotion, narrative engagement level, and word-of-mouth intention. J. Consum. Behav. 19, 47–56. doi: 10.1002/cb.1793

[ref74] Kim Y. K. Fingerman K. L. (2022). Daily social media use, social ties, and emotional well-being in later life. J. Soc. Pers. Relat. 39, 1794–1813. doi: 10.1177/02654075211067254, PMID: 37727534 PMC10508904

[ref75] Kline R. B. (2023). Principles and practice of structural equation modeling. 3rd Edn. Guilford publications: New York, NY, USA, 14.

[ref76] Laer T. Ruyter K. Visconti L. M. Wetzels M. (2014). The extended transportation-imagery model: a meta-analysis of the antecedents and consequences of consumers' narrative transportation. J. Consum. Res. 40, 797–817. doi: 10.1086/673383

[ref77] Lazarus R. S. (1991). Emotion and adaptation: Oxford University Press.

[ref78] Lee R. M. Robbins S. B. (1995). Measuring belongingness: the social connectedness and the social assurance scales. J. Couns. Psychol. 42, 232–241. doi: 10.1037/0022-0167.42.2.232

[ref79] Leist A. K. (2013). Social media use of older adults: a mini-review. Gerontology 59, 378–384. doi: 10.1159/000346818, PMID: 23594915

[ref80] Litwin H. Shiovitz-Ezra S. (2011). Social network type and subjective well-being in a national sample of older Americans. The Gerontologist 51, 379–388. doi: 10.1093/geront/gnq094, PMID: 21097553 PMC3095651

[ref81] Liu Z. Li Z. (2024). Relationships between digital engagement and the mental health of older adults: evidence from China. PLoS One 19:0308071. doi: 10.1371/journal.pone.0308071 PMC11302864 39106268

[ref82] Liu Y. Shrum L. J. (2002). What is interactivity and is it always such a good thing? Implications of definition, person, and situation for the influence of interactivity on advertising effectiveness. J. Advert. 31, 53–64. doi: 10.1080/00913367.2002.10673685

[ref83] Liu X. Zheng B. Liu H. (2022). Understanding the social media interactivity paradox: the effects of social media interactivity on communication quality, work interruptions, and job performance. Inf. Technol. People 35, 1805–1828. doi: 10.1108/ITP-12-2020-0845

[ref84] Lombard M. Ditton T. (1997). At the heart of it all: the concept of presence. J. Comput.-Mediat. Commun. 3, JCMC321. doi: 10.1111/j.1083-6101.1997.tb00072.x

[ref85] M D. Blanchard-Fields F. (2012). Linking process and outcome in the study of emotion and aging. Perspect. Psychol. Sci. 7, 3–17. doi: 10.1177/1745691611424750, PMID: 22888369 PMC3413281

[ref86] Mar R. A. Oatley K. (2008). The function of fiction is the abstraction and simulation of social experience. Perspect. Psychol. Sci. 3, 173–192. doi: 10.1111/j.1745-6924.2008.00073.x, PMID: 26158934

[ref87] MobTech. (2020). China Short video industry insight report 2020. Available online at: https://www.mob.com/mobdata/report/114 (Accessed August 19, 2025).

[ref88] Nabi R. L. Green M. C. (2014). The role of a narrative’s emotional flow in promoting persuasive outcomes. Media Psychol. 18, 137–162. doi: 10.1080/15213269.2014.912585

[ref89] Nabi R. L. Myrick J. G. (2019). Uplifting fear appeals: considering the role of hope in fear-based persuasive messages. Health Commun. 34, 463–474. doi: 10.1080/10410236.2017.1422847, PMID: 29313717

[ref90] National Academies of Sciences and Medicine (2020). Social isolation and loneliness in older adults: Opportunities for the health care system. DC: The National Academies Press. 32510896

[ref9001] National Bureau of Statistics of China (2021). Communiqué of the seventh national population census (No. 5).

[ref91] Nations, U. (2013). Department of Economic and Social Affairs, population division. World Population Ageing 2013, ST/ESA/SER.A/348.

[ref92] Nimrod G. (2020a). Aging well in the digital age: technology in processes of selective optimization with compensation. J. Gerontol. Ser. B 75, 2008–2017. doi: 10.1093/geronb/gbz111, PMID: 31504873

[ref93] Nimrod G. (2020b). Older audiences in the digital media environment. Inf. Commun. Soc. 23, 1902–1916. doi: 10.1080/1369118X.2016.1164740

[ref94] Nowland R. Necka E. A. Cacioppo J. T. (2018). Loneliness and social internet use: pathways to reconnection in a digital world? Perspect. Psychol. Sci. 13, 70–87. doi: 10.1177/1745691617713052, PMID: 28937910

[ref95] Nummenmaa L. Glerean E. Viinikainen M. Jääskeläinen I. P. Hari R. Sams M. (2012). Emotions promote social interaction by synchronizing brain activity across individuals. Proc. Natl. Acad. Sci. 109, 9599–9604. doi: 10.1073/pnas.1206095109, PMID: 22623534 PMC3386135

[ref96] Nunnally J. C. Bernstein I. H. (1994). The assessment of reliability. Psychometric theory, 3, 248–292.

[ref97] Oh H. J. Ozkaya E. LaRose R. (2014). How does online social networking enhance life satisfaction? The relationships among online supportive interaction, affect, perceived social support, sense of community, and life satisfaction. Comput. Hum. Behav. 30, 69–78. doi: 10.1016/j.chb.2013.07.053

[ref98] Páez D. Rimé B. Basabe N. Wlodarczyk A. Zumeta L. (2015). Psychosocial effects of perceived emotional synchrony in collective gatherings. J. Pers. Soc. Psychol. 108, 711–729. doi: 10.1037/pspi0000014, PMID: 25822033

[ref99] Panzarasa P. Griffiths C. J. Sastry N. De Simoni A. (2020). Social medical capital: how patients and caregivers can benefit from online social interactions. J. Med. Internet Res. 22:e16337. doi: 10.2196/16337, PMID: 32720910 PMC7420688

[ref100] Paúl C. Ribeiro O. Teixeira L. (2012). Active ageing: an empirical approach to the WHO model. Curr. Gerontol. Geriatr. Res. 2012:382972. doi: 10.1155/2012/382972, PMID: 23193396 PMC3501803

[ref101] Pinquart M. Sörensen S. (2000). Influences of socioeconomic status, social network, and competence on subjective well-being in later life: a meta-analysis. Psychol. Aging 15, 187–224. doi: 10.1037/0882-7974.15.2.187, PMID: 10879576

[ref102] Polkinghorne D. E. (1991). Narrative and self-concept. J. Narrative Life History 1, 135–153. doi: 10.1075/jnlh.1.2-3.04nar, PMID: 33486653

[ref103] Pressman S. D. Cohen S. (2005). Does positive affect influence health? Psychol. Bull. 131, 925–971. doi: 10.1037/0033-2909.131.6.925 16351329

[ref104] Putnam R. D. (2000). Bowling alone: The collapse and revival of American community: Simon & Schuster. doi: 10.1145/358916.361990

[ref105] Qin R. (2024). Miniature time: a study on the narrative and communication effects of online mini dramas from the perspective of empathy. Trans. Soc. Sci. Educ. Human. Res. 7, 163–171. doi: 10.62051/r8etm154

[ref106] Quan-Haase A. Mo G. Y. Wellman B. (2017). Connected seniors: how older adults in East York exchange social support online and offline. Inf. Commun. Soc. 20, 967–983. doi: 10.1080/1369118X.2017.1305428

[ref107] Results C. (2025). What do seniors do online? 2025 data for marketers. Creating Results. Available online at: https://creatingresults.com/blog/2025/03/13/what-do-seniors-do-online-2025-data-for-marketers/ (Accessed August 20, 2025).

[ref108] Reuters. (2024). Low-brow, vulgar’ micro-dramas shake up China’s film industry, aim for Hollywood. Reuters. Available online at: https://www.reuters.com/world/china/low-brow-vulgar-micro-dramas-shake-up-chinas-film-industry-aim-hollywood-2024-09-21/ (Accessed August 17, 2025).

[ref109] Rimé B. (2009). Emotion elicits the social sharing of emotion: theory and empirical review. Emot. Rev. 1, 60–85. doi: 10.1177/1754073908097189

[ref110] Rios Rincon A. M. Miguel Cruz A. Daum C. Neubauer N. Comeau A. Liu L. (2022). Digital storytelling in older adults with typical aging, and with mild cognitive impairment or dementia: a systematic literature review. J. Appl. Gerontol. 41, 867–880. doi: 10.1177/07334648211015456, PMID: 34009053 PMC8848055

[ref111] Rook K. S. (2015). Social networks in later life: weighing positive and negative effects on health and well-being. Curr. Dir. Psychol. Sci. 24, 45–51. doi: 10.1177/0963721414551364, PMID: 26366047 PMC4564251

[ref112] Rowe J. W. Kahn R. L. (1997). Successful aging. The Gerontologist 37, 433–440. doi: 10.1093/geront/37.4.433, PMID: 9279031

[ref113] Ryff C. D. Singer B. H. (2008). Know thyself and become what you are: a eudaimonic approach to psychological well-being. J. Happiness Stud. 9, 13–39. doi: 10.1007/s10902-006-9019-0

[ref114] Sarstedt M. Ringle C. M. Henseler J. Hair J. F. (2014). On the emancipation of PLS-SEM: a commentary on Rigdon (2012). Long Range Plan. 47, 154–160. doi: 10.1016/j.lrp.2014.02.007

[ref115] Scheibe K. Zimmer F. Fietkiewicz K. J. Stock W. G. (2022). Interpersonal relations and social actions on live streaming services: a systematic review on cyber-social relations.

[ref116] Scherer K. R. (2001). “Appraisal considered as a process of multilevel sequential checking” in Appraisal processes in emotion: Theory, methods, research. eds. Scherer K. R. Schorr A. Johnstone T. (Oxford University Press), 92–120.

[ref117] Sen K. Prybutok G. Prybutok V. (2021). The use of digital technology for social wellbeing reduces social isolation in older adults: a systematic review. SSM Popul. Health 17:101020. doi: 10.1016/j.ssmph.2021.101020 35024424 PMC8733322

[ref118] Short J. Williams E. Christie B. (1976). The social psychology of telecommunications. Hoboken: Wiley.

[ref119] Silverstein M. Parker M. G. (2002). Leisure activities and quality of life among the oldest old in Sweden. Res. Aging 24, 528–547. doi: 10.1177/0164027502245003

[ref120] Slater M. D. Johnson B. K. Cohen J. Comello M. L. G. Ewoldsen D. R. (2014). Temporarily expanding the boundaries of the self: motivations for entering the story world and implications for narrative effects. J. Commun. 64, 439–455. doi: 10.1111/jcom.12100

[ref121] Slater M. D. Rouner D. (2002). Entertainment–education and elaboration likelihood: understanding the processing of narrative persuasion. Commun. Theory 12, 173–191. doi: 10.1111/j.1468-2885.2002.tb00265.x, PMID: 41131858

[ref122] Stargatt J. Bhar S. Bhowmik J. Al Mahmud A. (2022). Digital storytelling for health-related outcomes in older adults: systematic review. J. Med. Internet Res. 24:28113. doi: 10.2196/28113, PMID: 35019845 PMC8792772

[ref123] Sundar S. S. (2008a). The MAIN model: a heuristic approach to understanding technology effects on credibility. In Digital media, youth, and credibility; Metzger M.J. Flanagin A.J., Eds., 73–100. doi: 10.1162/dmal.9780262562324.073

[ref124] Sundar S. S. (2008b). The MAIN model: a heuristic approach to understanding technology effects. J. Broadcast. Electron. Media 57, 504–525.

[ref125] Sundar S. S. Limperos A. M. (2013). Uses and gratifications 2.0: new gratifications for new media. J. Broadcast. Electron. Media 57, 504–525. doi: 10.1080/08838151.2013.845827

[ref126] Tang X. Ding X. Zhou Z. (2023). Towards equitable online participation: a case of older adult content creators' role transition on short-form video sharing platforms. Proc. ACM Hum.-Comp. Interact. 7, 1–22. doi: 10.1145/3610216

[ref127] Trowbridge J. (1976). The uses of mass communications: current perspectives on gratifications research by Blumler Jay G. Katz Elihu, American Journal of Sociology 81, 1546–1548.

[ref128] Tsai Y. I. Beh J. Ganderton C. Pranata A. (2024). Digital interventions for healthy ageing and cognitive health in older adults: a systematic review of mixed-method studies and meta-analysis. BMC Geriatr. 24:217. doi: 10.1186/s12877-023-04617-3, PMID: 38438870 PMC10910826

[ref129] Tukachinsky R. Stever G. (2019). Theorizing development of parasocial engagement. Commun. Theory 29, 209–230. doi: 10.1093/ct/qty032, PMID: 40881048

[ref130] Uchino B. N. (2006). Social support and health: a review of physiological processes potentially underlying links to disease outcomes. J. Behav. Med. 29, 377–387. doi: 10.1007/s10865-006-9056-5, PMID: 16758315

[ref131] Vorderer P. Klimmt C. Ritterfeld U. (2004). Enjoyment: at the heart of media entertainment. Commun. Theory 14, 388–408. doi: 10.1111/j.1468-2885.2004.tb00321.x

[ref132] Williams A. Nussbaum J. F. (2013). Intergenerational communication across the life span: Routledge.

[ref133] Wohn D. Y. Lampe C. Wash R. Ellison N. Vitak J. (2011). “The "S" in social network games: initiating, maintaining, and enhancing relationships.” In Proceedings of the 44th Annual Hawaii International Conference on System Sciences,

[ref134] World Health Organization (2002). Active ageing: a policy framework: World Health Organization. Avilable online at: https://extranet.who.int/agefriendlyworld/active-ageing-a-policy-framework/ (Accessed 20 August, 2025).

[ref135] World Health Organization. (2023). Ageing and health. Retrieved 7–24. Available online at: https://www.who.int/news-room/fact-sheets/detail/ageing-and-health (Accessed August 19, 2025).

[ref136] Wu X. (2024). Some advice for China's elderly regarding the use of mobile banking. Econ. Soc. Human. 1, 7–11. doi: 10.62381/E244202

[ref137] Wu Z. Wen Q. (2023). Research on the current policy system for coping with the problem of population ageing. Aging Res. 10:77353. doi: 10.12677/AR.2023.104194

[ref138] Xu L. Fields N. L. Daniel K. M. Cipher D. J. Troutman B. A. (2023). Reminiscence and digital storytelling to improve the social and emotional well-being of older adults with Alzheimer's disease and related dementias: protocol for a mixed methods study design and a randomized controlled trial. JMIR Res. Protocols 12:e49752. doi: 10.2196/49752, PMID: 37676706 PMC10514775

[ref139] Yen H. Y. Chi M. J. Huang H. Y. (2022). Social engagement for mental health: an international survey of older populations. Int. Nurs. Rev. 69, 359–368. doi: 10.1111/inr.12737, PMID: 34874057

[ref140] Yu X. Mu A. Wu X. Zhou L. (2022). Impact of internet use on cognitive decline in middle-aged and older adults in China: longitudinal observational study. J. Med. Internet Res. 24:e25760. doi: 10.2196/25760, PMID: 35072642 PMC8822429

[ref141] Yuan X. (2024). China’s ‘ultrashort’ dramas embrace the mid-life crisis. Sixth Tone. Available online at: https://www.sixthtone.com/news/1016167?utm_source=chatgpt.com (Accessed August 20, 2025).

[ref142] Zhang K. Kim K. Silverstein N. M. Song Q. Burr J. A. (2021). Social media communication and loneliness among older adults: the mediating roles of social support and social contact. The Gerontologist 61, 888–896. doi: 10.1093/geront/gnaa197, PMID: 33284972

[ref143] Zhang R. Su Y. Lin Z. Hu X. (2024). The impact of short video usage on the mental health of elderly people. BMC Psychology 12:612. doi: 10.1186/s40359-024-02125-6, PMID: 39482710 PMC11529554

[ref144] Zhou Y. Bai Y. Wang J. (2024). The impact of internet use on health among older adults in China: a nationally representative study. BMC Public Health 24:1065. doi: 10.1186/s12889-024-18269-4, PMID: 38632588 PMC11022463

[ref145] Zhou F. Zhou X. (2022). Across time and space, I am together with many, many others: digital writing and temporality on Chinese social media. Soc. Media Soc. 8:7564. doi: 10.1177/20563051221117564

[ref146] Zuo S. Wang Y. (2023). Research on functional design of online social platforms for older adults. Operat. Res. Fuzziol. 13, 593–601. doi: 10.12677/orf.2023.132059

