# Supplementary material for: Impact of content features of digital micro-dramas on active aging: the serial mediation effect of emotional engagement and social connectedness
Source: Front Psychol. 2025 Nov 7;16:1711672. doi: 10.3389/fpsyg.2025.1711672 (PMC12634578; doi:10.3389/fpsyg.2025.1711672)
Supplement: Supplementary file 1 [file Supplementary_file_1.pdf]

Appendix A. Questionnaire

Impact of Content Features of Digital Micro-Dramas on Active Aging: The Serial Mediation of Emotional Engagement and Social Connectedness

Survey Instructions

Dear Participant,

This academic survey aims to explore how the content features of digital micro-dramas influence emotional and social experiences among older adults, thereby promoting active aging.

Please read each statement carefully and select the option that best reflects your personal opinion or experience.

The questionnaire is anonymous, and all responses will be used for academic purposes only.

There are no right or wrong answers. Please choose according to your genuine perception.

Unless otherwise stated, all items adopt a 7-point Likert scale:

1 = Strongly Disagree 2 = Disagree 3 = Somewhat Disagree 4 = Neutral 5 = Somewhat Agree 6 = Agree 7 = Strongly Agree.

Estimated completion time: approximately 8–10 minutes.

Thank you for your participation!

Section I. Demographic Information

1．**Age:** 60–65 □ 65–70 □ 70–75 □ 75–80 □ 80–85 □ 85+ □

2．**Gender:** Male □ Female □

3．**Education Level:** Primary □ Junior High □ High School □ Associate Degree □ Bachelor’s □ Postgraduate □

4．**Marital Status:** Single □ Married □ Widowed □ Divorced □ Other

5．**Living Arrangement:** Alone □ With spouse □ With children □ With relatives/friends □ Institution □ Other □

6．**Self-rated Health:** (1 = Very poor … 7 = Excellent) ____

7．**Digital Literacy (self-rated)**: (1 = Very low … 7 = Very high) ____

8．**Viewing Frequency of Micro-Dramas:** Rarely □ 1–2 days/week □ 3–4 days/week □ Almost daily □ Multiple times/day □

9．**Average Viewing Duration per Day**: <10 min □ 10–30 min □ 31–60 min □ 61–120 min □ >120 min □

10．**Preferred Platforms (multiple choices):** Douyin □ Kuaishou □ Bilibili □ WeChat Video □ YouTube □ Other ________

11．**Favorite Genres:** Family/Emotion □ Career/Retirement □ Health/Care □ Community Life □ Other ________

Section II. Main Constructs (7-Point Likert Scale)

1．Narrativity (NA)

NA1: These digital micro-dramas have a compact storyline with a clear structure.

NA2: The story structure is coherent and complete, flowing naturally, which makes it easy for me to understand.

NA3: The story often features unexpected plot twists.

NA4: The beginning of the story is highly engaging, making me eager to continue watching.

2. Emotional Resonance (ER)

ER1: The beginning of the story is highly engaging, making me eager to continue watching.

ER2: These contents frequently bring me feelings of joy or pleasure.

ER3: I tend to empathize with the characters in the story (feeling nervous, happy, etc., for them).

ER4: After watching, I often find myself reflecting on the plot or thinking about its meaning.

3. Interactivity (IN)

IN1: I can easily comment, like, send bullet comments, or interact through private messages.

IN2: I use interactive features, such as branching story choices, autoplay, and speed adjustments.

IN3: I can easily use the platform's sharing function to interact.

IN4: I participate in interactive activities on the platform's community, such as storyline voting, discussions, and creating derivative content.

4. Emotional Engagement (EE)

EE1: While watching, I often immerse myself completely and care about the development of the plot.

EE2: The storyline makes me experience emotional fluctuations (such as tension, anticipation, surprise).

EE3: I develop an emotional attachment to my favorite characters or scenes.

EE4: I look forward to the next episode or seek out similar micro-dramas to watch.

5. Social Connectedness (SC)

SC1: By watching micro-dramas, I have made new friends or expanded my social circle.

SC2: I can receive support and responses from others in the comment section or group chats.

SC3: I have more topics to discuss with people who share similar interests, and we interact frequently.

SC4: My online interactions often extend to offline social activities.

6. Active Aging (AA)

AA1: Recently, I have become more proactive about health management and life goals.

AA2: I enjoy participating in family, community, or online activities.

AA3: Lately, I am more willing to learn digital skills (such as mobile payments, video calls) to maintain social connectedness.

AA4: I am more motivated to make long-term life plans (such as travel, volunteer work).
